# LINC01431 Promotes Histone H4R3 Methylation to Impede HBV Covalently Closed Circular DNA Transcription by Stabilizing PRMT1

**DOI:** 10.1002/advs.202103135

**Published:** 2022-04-10

**Authors:** Yang Sun, Yan Teng, Liyuan Wang, Zhaoying Zhang, ChaoJia Chen, Yingchun Wang, Xiaodong Zhang, Peng Xiang, Xiaojia Song, Jinghui Lu, Nailin Li, Lifen Gao, Xiaohong Liang, Yuchen Xia, Zhuanchang Wu, Chunhong Ma

**Affiliations:** ^1^ Key Laboratory for Experimental Teratology of Ministry of Education and Department of Immunology School of Basic Medical Sciences Cheeloo Medical College Shandong University Jinan Shandong 250012 China; ^2^ State Key Laboratory of Virology and Hubei Province Key Laboratory of Allergy and Immunology Institute of Medical Virology School of Basic Medical Sciences Wuhan University Wuhan Hubei 430072 China; ^3^ Department of Hepatobiliary Surgery Qilu Hospital of Shandong University, Jinan Shandong 250012 China; ^4^ Karolinska Institute Department of Medicine‐Solna Clinical Pharmacology Group Stockholm 17176 Sweden

**Keywords:** Epigenetics, HBV transcription, Histone methylation, LncRNA, PRMT1

## Abstract

Covalently closed circular DNA (cccDNA) is the transcriptional template of hepatitis B virus (HBV), which interacts with both host and viral proteins to form minichromosome in the nucleus and is resistant to antiviral agents. Identification of host factors involved in cccDNA transcriptional regulation is expected to prove a new venue for HBV therapy. Recent evidence suggests the involvement of long noncoding RNAs (lncRNAs) in mediating the interaction of host factors with various viruses, however, lncRNAs that HBV targets and represses cccDNA transcription have not been fully elucidated. Here, the authors identified LINC01431 as a novel host restriction factor for HBV transcription. Mechanically, LINC01431 competitively bound with type I protein arginine methyltransferase (PRMT1) to block the HBx‐mediated PRMT1 ubiquitination and degradation. Consequently, LINC01431 increased the occupancy of PRMT1 on cccDNA, leading to enhanced H4R3me2a modification and reduced acetylation of cccDNA‐bound histones, thereby repressing cccDNA transcription. In turn, to facilitate viral replication, HBV transcriptionally repressed LINC01431 expression by HBx‐mediated repression of transcription factor Zinc fingers and homeoboxes 2 (ZHX2). Collectively, the study demonstrates LINC01431 as a novel epigenetic regulator of cccDNA minichromosome and highlights a feedback loop of HBx‐LINC01431‐PRMT1 in HBV replication, which provides potential therapeutic targets for HBV treatment.

## Graphical abstract

LINC01431 is identified as a novel host restriction factor for HBV replication. Mechanically, LINC01431 stabilizes PRMT1 and increases its occupancy on cccDNA, leading to enhanced H4R3me2a modification and reduced acetylation of cccDNA‐bound histones, thereby repressing cccDNA transcription. In turn, HBx represses LINC01431 expression by targeting ZHX2. Collectively, the authors’ study presents a novel HBx‐LINC01431‐PRMT1 feedback loop for regulating HBV replication.

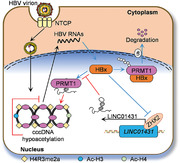

## 1 Introduction

Hepatitis B virus (HBV) infection remains a heavy burden for common health worldwide.^[^ ^1^, ^2^ ^]^ Although the new incidence of HBV infection has been successfully reduced by protective vaccination,^[^ ^3^ ^]^ chronic HBV infection accounts for about half of all hepatocellular carcinoma (HCC) cases and about 33% of total HCC deaths observed.^[^ ^4^, ^5^ ^]^ Current treatments for chronic HBV infection are limited to type I interferon and nucleotide analogues, both of which are not curative because they have limited effect on the viral replication template, covalently closed circular DNA (cccDNA).^[^ ^6^ ^]^

HBV is a relaxed circular, partially double‐stranded DNA virus with a genome of 3.2 kb that encodes four overlapped open reading frames (ORFs).^[^ ^7^ ^]^ Upon infection, HBV relaxed circular DNA (rcDNA) is converted to cccDNA. cccDNA accumulates in the nucleus and interacts with histones and non‐histones to form minichromosome, which serves as the template to achieve viral RNA transcription.^[^ ^8^, ^9^ ^]^ It is clear that a series of histone posttranslational modifications (PTMs), including active PTMs H3K4me3, H3K27ac, H3K36me3, H3K122ac, as well as repressive PTMs H3K9me3 and H3K27me3, are directly enriched on cccDNA, providing dynamic epigenetic modulation of cccDNA transcriptional activity.^[^ ^10^, ^11^ ^]^ Recent studies suggest that these epigenetic machineries have been adopted by HBx, the most critical viral protein, to promote HBV transcription. For instance, HBx recruits p300/CBP and PACF to cccDNA and acetylates histones to promote HBV replication.^[^ ^9^ ^]^ Also, HBx transactivates HAT1 and promotes histones acetylation on cccDNA to enhance cccDNA transcription.^[^ ^2^ ^]^ However, host factors that are both involved in cccDNA transcriptional regulation and manipulated by HBV/HBx have not been fully identified.^[^ ^2^, ^12^ ^]^ Further identification of such host factors is required for defining drug targets for HBV treatment.

Long noncoding RNAs (lncRNAs) are a heterogeneous class of transcripts that are larger than 200 nucleotides with little or no protein‐coding potential and have been shown to exert critical regulatory roles in various cellular processes including modulating gene expression and epigenetic modifications.^[^ ^2^, ^13^ ^]^ Emerging evidence has suggested the involvement of lncRNAs in mediating the interaction between host cells and multiple viruses including HCV and HBV.^[^ ^14^, ^15^ ^]^ And lncRNAs have been reported to regulate HBV replication by remodeling cccDNA minichromosome in an HBx‐dependent manner.^[^ ^2^ ^]^ LncRNA HULC activates HBV replication and accelerates HCC progress by modulating HBx/STAT3/miR‐539/APOBEC3B signal pathway.^[^ ^16^ ^]^ LncRNA HOTAIR interacts with DDX5/PRC2 to repress cccDNA transcription,^[^ ^17^ ^]^ while HBx‐bound lncRNA DLEU2 sustains transcription of cccDNA and cancer‐related genes.^[^ ^12^ ^]^ Therefore, identification of novel lncRNAs, which are targeted by HBx and epigenetically repress HBV transcription, is important for developing new HBV therapeutic strategies.

In this study, we identify LINC01431 as a novel HBV restriction lncRNA that is downregulated by HBx and represses cccDNA transcription. Our data reveal that LINC01431 interacts with and enhances the stability of type I protein arginine methyltransferase (PRMT1), an epigenetic remodeler of cccDNA minichromosome. HBx represses LINC01431 transcription and decreases the stability of PRMT1, resulting in reduced PRMT1 enrichment on cccDNA, therefore enhancing cccDNA accessibility and transcription. Our findings underscore the crucial regulatory role of HBx‐LINC01431‐PRMT1 in regulating HBV cccDNA transcription, which may serve as a potential therapeutic target.

## 2 Results

### 2.1 LINC01431 Is a Novel lncRNA that Is Downregulated by HBx during HBV Infection

**Figure 1:**
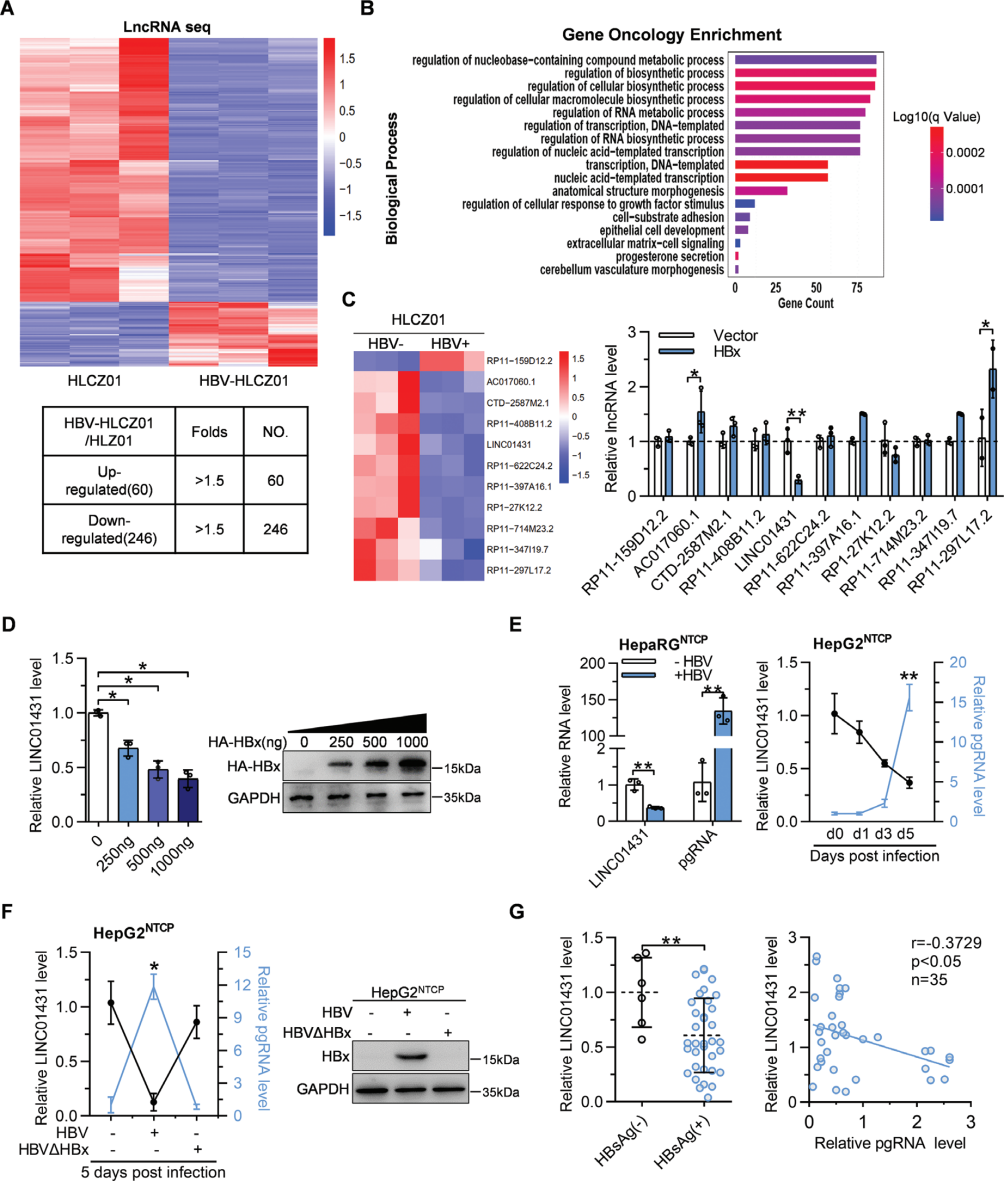
HBV infection especially HBx downregulates LINC01431 expression. A–C) LncRNA sequencing was performed in HLCZ01 cells with or without HBV infection. A) The heat map of lncRNA sequencing was shown in the upper panel and the number of differentially expressed lncRNAs was shown in the bottom panel. Upregulated and downregulated lncRNAs were colored in red and blue, respectively. B) Gene Ontology analysis focused on biological processes after HBV infection, summarized based on the enrichment score. C) The heat map of 11 novel lncRNAs related to transcriptional regulation and RNA synthesis was shown in the left panel, and the expression levels of these lncRNAs in HBx‐transfected Huh7 cells detected using RT‐qPCR was shown in the right panel. D) RT‐qPCR and immunoblot analysis of LINC01431 RNA and HBx expression in HBx‐transfected Huh7 cells. E) RT‐qPCR analysis of LINC01431 and pgRNA in HBV‐infected HepaRG^NTCP^ (left) and HepG2^NTCP^ cells (right). F) Expression of LINC01431, pgRNA and HBx in HBV and mutant HBVΔHBx‐infected HepG2^NTCP^ cells. The total RNA and protein were extracted at 5 days post infection (dpi), followed by RT‐qPCR and immunoblotting, respectively. G) Correlation analysis of LINC01431 with HBsAg (left) and pgRNA (right) in human HCC para‐tumor tissues. The samples were divided into HBsAg negative and HBsAg positive groups according to the presence of HBsAg, and the levels of LINC01431 RNA were quantified by RT‐qPCR (left, HBsAg(−) *n* = 6, HBsAg(+) *n* = 35). For (C–F), representative of 3 independent experiments. Data information: data were presented as mean ± SD and normalized to the control group. Pearson's correlation coefficient (E–G). Two‐tail unpaired Student's *t*‐tests; ^*^ *p* < 0.05; ^**^ *p* < 0.01 (C–G).

To identify lncRNAs responsible for regulating HBV replication in hepatocytes, lncRNA sequencing was performed in HLCZ01 cells, a hepatoma cell line that supports the entire life cycle of HBV^[^ ^18^ ^]^ with or without HBV infection (Figure S1A, Supporting Information). Totally, 306 differentially expressed lncRNAs (Log_2_ Fold change >1.5; *p* < 0.05; 60 upregulated and 246 downregulated) were identified in HBV‐infected HLCZ01 cells (**Figure** **1**A). Gene Ontology enrichment analysis of putative targets of lncRNAs^[^ ^19^ ^]^ suggested the significant enrichment of transcriptional regulation and RNA synthesis‐related biological processes in HBV‐infected HLCZ01 cells (Figure 1B). Further analysis showed that 11 novel lncRNAs with unknown functions were significantly dysregulated in HBV‐infected HLCZ01 cells (Figure 1C left). Considering HBx serving as the predominate viral protein which is critically involved in regulating host factors to promote HBV transcription,^[^ ^20^, ^21^ ^]^ we then investigated the expression of these novel lncRNAs regulated by HBx during HBV infection. As shown in Figure 1C right and Figure S1B, Supporting Information, among 11 lncRNAs, LINC01431 expression was significantly inhibited in HBx‐overexpressed Huh7 cells and also showed a dynamic decrease in HBV‐infected Huh7^NTCP^ cells. Moreover, HBx repressed LINC01431 expression in a dose‐dependent manner (Figure 1D). We further confirmed the HBV‐mediated downregulation of LINC01431 in pHBV1.3‐transfected Huh7 cells and HBV‐infected HepaRG^NTCP^, HepG2^NTCP^ cells and HLCZ01 cells (Figure 1E and Figure S1C, Supporting Information). More importantly, lack of HBx (ATG→TTG mutation causing HBx deletion, HBV∆HBx) in HBV‐infected HepG2^NTCP^ cells and pHBV1.1‐transfected HepG2 cells almost wholly destroyed the HBV‐induced suppression of LINC01431 (Figure 1F and Figure S1D, Supporting Information). Consistently, RT‐qPCR analysis confirmed the significantly decreased expression of LINC01431 in HCC para‐tumor tissues from HBV‐positive HCC patients than that from HBV‐negative patients, and the levels of LINC01431 showed a significantly negative correlation with the levels of pregenomic RNA (pgRNA) in HBV‐positive HCC patients (Figure 1G). Collectively, these findings reveal that LINC01431 is a novel lncRNA downregulated by HBx, and that the high level of HBV replication is associated with the low level of LINC01431.

### 2.2 LINC01431 Is a Nuclear lncRNA which Represses HBV cccDNA Transcription

**Figure 2:**
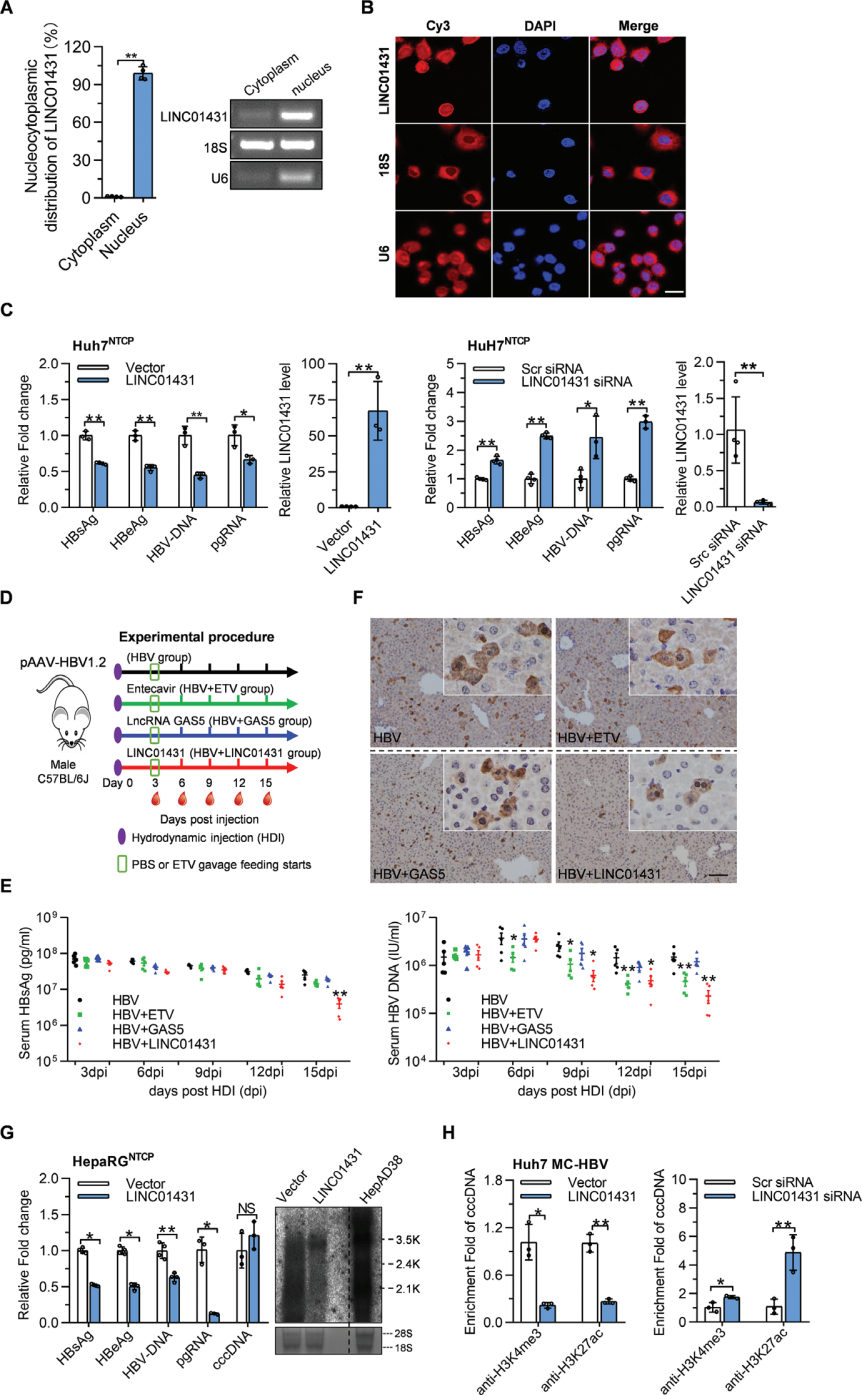
LINC01431 is localized in the nucleus and represses HBV cccDNA transcription. A) RT‐qPCR analysis of LINC01431 RNA in the cytoplasmic and nuclear fraction of HepG2 cells. B) RNA FISH assays were performed in Huh7 cells. Nuclei were stained with DAPI. Scale bar, 20 µm. C) LINC01431 overexpression and knockdown were performed in HBV‐infected Huh7^NTCP^ cells. The levels of HBV antigens, HBV‐DNA, and pgRNA were detected at 5 dpi. D,F) HBV carrier mice were prepared by hydrodynamic injection (HDI) of pAAV‐HBV1.2 and then were randomly divided into four groups as indicated. Blood was collected every 3 days, and mice were sacrificed at 15 dpi. The levels of HBsAg, HBV‐DNA, and HBcAg were measured by ELISA, IHC, and RT‐qPCR, respectively. Animal study design (D). HBsAg and HBV‐DNA in serum (E), and hepatic HBcAg (F) were measured (*n* = 5).Scale bar, 50 µm. G) LINC01431 overexpression was performed in HBV‐infected HepaRG^NTCP^ cells, and the levels of HBV antigens, HBV‐DNA, cccDNA, and HBV RNAs were detected at 7 dpi. H) ChIP assay was performed in MC‐HBV‐transfected Huh7 cells with the presence of LINC01431 (left) or LINC01431 siRNA (right). The enrichment of H3K4me3 and H3K27ac on cccDNA was determined at day 3 post transfection. Normalized data were shown as relative fold enrichment to the control group. For all experiments, representative of 3 independent experiments. Data information: data were presented as mean ± SD and normalized to the control group. Two‐tail unpaired Student's *t*‐tests; ^*^ *p* < 0.05; ^**^ *p* < 0.01; *NS*, no significance (A,C,E,F–H).

*LINC01431* is an intergenic lncRNA located on chromosome 20p11.21, and the full‐length transcript is 647 nt (Figure S1E, Supporting Information). Since the subcellular localization of lncRNAs is the primary determinant of their molecular functions,^[^ ^22^ ^]^ we then separated the cytoplasmic and nuclear fractions of HepG2 cells and detected LINC01431 expression. As shown in **Figure** **2**A, LINC01431 was localized in the nucleus. And the nuclear localization of LINC01431 was further confirmed using RNA fluorescence in situ hybridization (RNA FISH) in Huh7 cells (Figure 2B).

To evaluate the role of LINC01431 in HBV replication, LINC01431 overexpression and knockdown were performed in various HBV supporting cell models, including HBV‐infected Huh7^NTCP^ cells/HLCZ01 cells,^[^ ^18^, ^23^ ^]^ and HepG2/Huh7 cells transfected with pHBV1.3 or engineered minicircle HBV cccDNA (MC‐HBV) which forms authentic cccDNA‐like molecules to support viral replication in hepatocytes.^[^ ^24^, ^25^ ^]^ As shown in Figure 2C and Figure S2A–C, Supporting Information, LINC01431 overexpression significantly inhibited, while LINC01431 knockdown promoted HBV replication, displaying as increased production of HBV antigens (HBsAg/HBeAg), HBV virion, and pgRNA in all detected cells.

To further confirm the role of LINC01431 against HBV in vivo, LINC01431‐PT3EF1*α* plasmid was injected into HBV carrier mice prepared by pAAV‐HBV1.2 hydrodynamic injection (HDI), the nucleotide analogues Entecavir (ETV) was selected as a positive control, and lncRNA GAS5, which showed no role in HBV replication,^[^ ^26^ ^]^ as a negative control (Figure 2D and Figure S2D, Supporting Information). As expected, compared with GAS5, LINC01431 overexpression significantly reduced the serum levels of HBsAg and HBV DNA, as well as hepatic HBcAg expression, while ETV treatment significantly decreased the levels of serum HBV‐DNA but not HBsAg (Figure 2E,F and Figure S2E, Supporting Information). Collectively, the above findings demonstrate LINC01431 as a host restriction factor for HBV replication.

To characterize the LINC01431‐primed HBV inhibition in detail, several cell models supporting HBV replication were employed. LINC01431 overexpression significantly decreased HBV antigens, viral DNA, and RNAs in HepaRG^NTCP^ cells (Figure 2G), but had little effect on the level of cccDNA and the stability of pgRNA (Figure 2G and Figure S2F,G, Supporting Information), suggesting that LINC01431 specifically repressed cccDNA transcription. In accordance, luciferase reporter assays showed that LINC01431 overexpression decreased the transcriptional activity of HBV promoters, and LINC01431 inhibited the transcriptional activity of HBx promoter in a dose‐dependent manner (Figure S2H, Supporting Information). Given that histones modifications on cccDNA minichromosome play a crucial role in regulating cccDNA transcription,^[^ ^10^ ^]^ we then explored whether LINC01431 affected the epigenetic modifications of cccDNA. Interestingly, results of chromatin immunoprecipitation (ChIP) assay demonstrated that LINC01431 overexpression greatly decreased the enrichment of active modifications including H3K4me3 and H3K27ac, while LINC01431 knockdown markedly increased these modifications on cccDNA‐bound histones (Figure 2H). These data suggest that LINC01431 is a novel lncRNA that modulates histone modifications on cccDNA and restricts HBV transcription.

### 2.3 PRMT1 Is the Binding Partner of LINC01431 which Mediates the Inhibition of LINC01431 on HBV Replication

**Figure 3:**
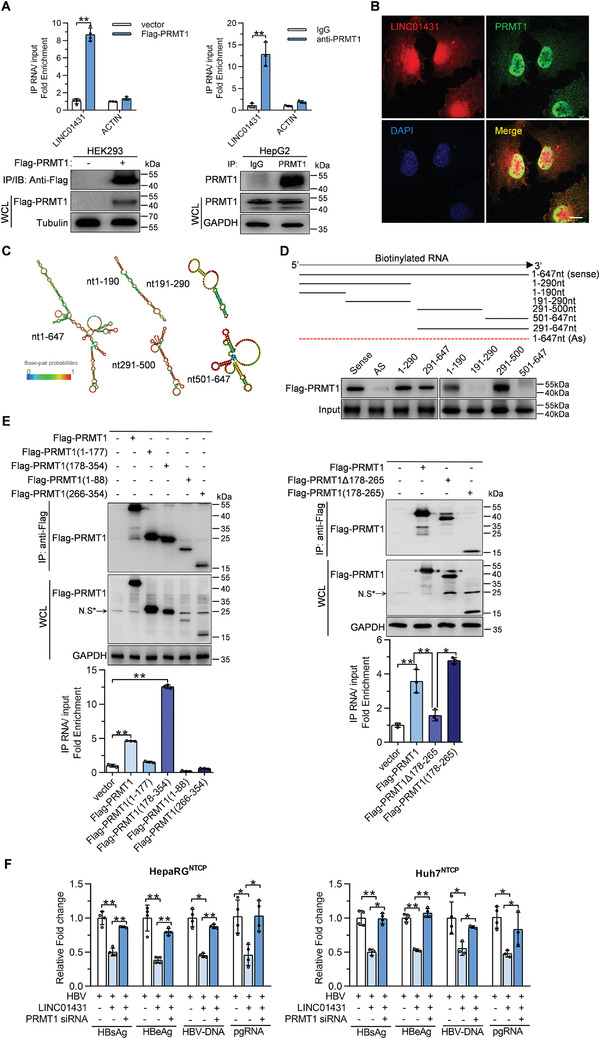
PRMT1 interacts with LINC01431 to mediate its anti‐HBV effect. A) RIP assay for LINC01431 in Flag‐PRMT1 overexpressed HEK293 and HepG2 cells using anti‐Flag (left) or anti‐PRMT1 (right). Normalized data were shown as relative fold enrichment to the control group. B) Immunofluorescence of LINC01431 and PRMT1 in Huh7 cells. Nuclei were stained with DAPI (blue). Scale bar, 20 µm. C) Predicted secondary structures of LINC01431 and its truncated mutants were presented. D) RNA‐pull down assays for the in vitro interaction between truncated LINC01431 mutants and PRMT1. Huh7 cells were transfected with Flag‐PRMT1, and the cell lysates were incubated with biotinylated LINC01431 transcripts, followed by immunoblotting with indicated antibodies. E) RIP assay for the in vivo interaction between LINC01431 and PRMT1. HepG2 cells were transfected with LINC01431 and plasmids expressing different Flag‐tagged‐PRMT1 truncates. RIP assay was performed using anti‐Flag antibody at day 3 post transfection. The level of PRMT1 and its mutants were detected by immunoblotting (upper). Normalized data were shown as relative fold enrichment to the control group (bottom). N.S* indicated the non‐specific band. F) Rescue assays were performed in HBV‐infected HepaRG^NTCP^ cells (left) or HBV‐infected Huh7^NTCP^ cells (right) after silencing PRMT1. The levels of HBV antigens, HBV‐DNA, and pgRNA were detected at 5 dpi by ELISA and RT‐qPCR, respectively. WCL indicated the whole cell lysis. GAPDH and Tubulin served as the loading control. For (A,B) and (D–F), representative of 3 independent experiments. Data information: data were presented as mean ± SD and normalized to the control group. One‐way ANOVA (F). Two‐tail unpaired Student's *t*‐tests; ^*^ *p* < 0.05; ^**^ *p* < 0.01 (A,E).

To investigate the mechanism by which LINC01431 restricts cccDNA transcription, RNA‐pull down coupled with mass spectrometry assay was performed. In vitro‐transcribed LINC01431 was successfully biotinylated and incubated with Huh7 cells nuclear extracts for RNA‐pull down assay, with biotinylated LINC01431 antisense transcript as a negative control (Figure S3A, Supporting Information). Silver staining analysis specifically detected a band of ≈40 kDa in the LINC01431‐sense group, which was cut off and subjected to mass spectrometry. ENO1, PRMT1, PCBP2, and RACK1 were the top 4 identified proteins and were selected as the potential targets (Figure S3A, Supporting Information). Among these, PRMT1, a type I protein arginine methyltransferase previously reported as a negative regulator of HBV replication,^[^ ^27^ ^]^ was the only validated binding partner of LINC01431 (Figure S3B, Supporting Information). In accordance, RNA immunoprecipitation (RIP) assays in Flag‐PRMT1‐overexpressed HEK293 cells demonstrated the significant enrichment of LINC01431 in PRMT1 (**Figure** **3**A left). The endogenous LINC01431‐PRMT1 interaction was also confirmed in HepG2 cells by RIP with anti‐PRMT1 antibody (Figure 3A right). In addition, immunofluorescence assays further verified the colocalization of LINC01431 and PRMT1 in Huh7 cells (Figure 3B).

To map the critical segment of LINC01431 that interacts with PRMT1, LINC01431 structure was predicted using the online RNA structure prediction software (http://rna.tbi.univie.ac.at/cgi‐bin/RNAWebSuite/RNAfold.cgi), and the mimetic diagrams are shown in Figure 3C. Accordingly, the LINC01431 truncated mutants were constructed basing on their secondary structure. RNA‐pull down assays demonstrated that nucleotides 1–190 or 291–500 of LINC01431, which formed a flat stem‐loop structure, were required to interact with PRMT1 in Huh7 cells (Figure 3D). Concurrently, further efforts were also made to define the critical region of PRMT1 that is responsible for the interaction with LINC01431. As shown in Figure 3E, full‐length PRMT1 and PRMT1(178‐354) showed significant enrichment with LINC01431 by RIP assays (Figure 3E left). Further truncation analysis confirmed that the 178–265 domain of PRMT1 effectively bound with LINC01431, and that mutant PRMT1 with 178–265 domain deletion (PRMT1(Δ178‐265)) lost its capability to occupy with LINC01431 (Figure 3E right).

The interaction of LINC01431 and PRMT1 inspired us to hypothesize that PRMT1 might be responsible for LINC01431‐mediated HBV inhibition. To address the hypothesis, PRMT1 was knockdown in hepatocytes harboring HBV and ectopic LINC01431. RT‐qPCR and immunoblot analysis confirmed the PRMT1 knockdown efficiency, respectively (Figure S3C, Supporting Information). As shown in Figure 3F, knockdown of PRMT1 almost entirely rescued LINC01431‐initiated HBV inhibition in HBV‐infected HepaRG^NTCP^ and Huh7^NTCP^ cells, displaying as elevated levels of HBV antigens, HBV‐DNA, and pgRNA. Similar results were obtained in pHBV1.3‐ and MC‐HBV‐transfected HepG2 cells (Figure S3D, Supporting Information). Furthermore, treatment with the PRMT1 inhibitor C‐7280948 markedly rescued the levels of HBV antigens, HBV‐DNA, and pgRNA in LINC01431‐overexpressed HepG2 cells (Figure S3E, Supporting Information). Together, these results suggest that PRMT1 is the binding partner of LINC01431, which mediates the inhibition of LINC01431 on HBV replication via its methyltransferase activity.

### 2.4 LINC01431 Enhances PRMT1 Protein Stability by Blocking PRMT1 Ubiquitination

**Figure 4:**
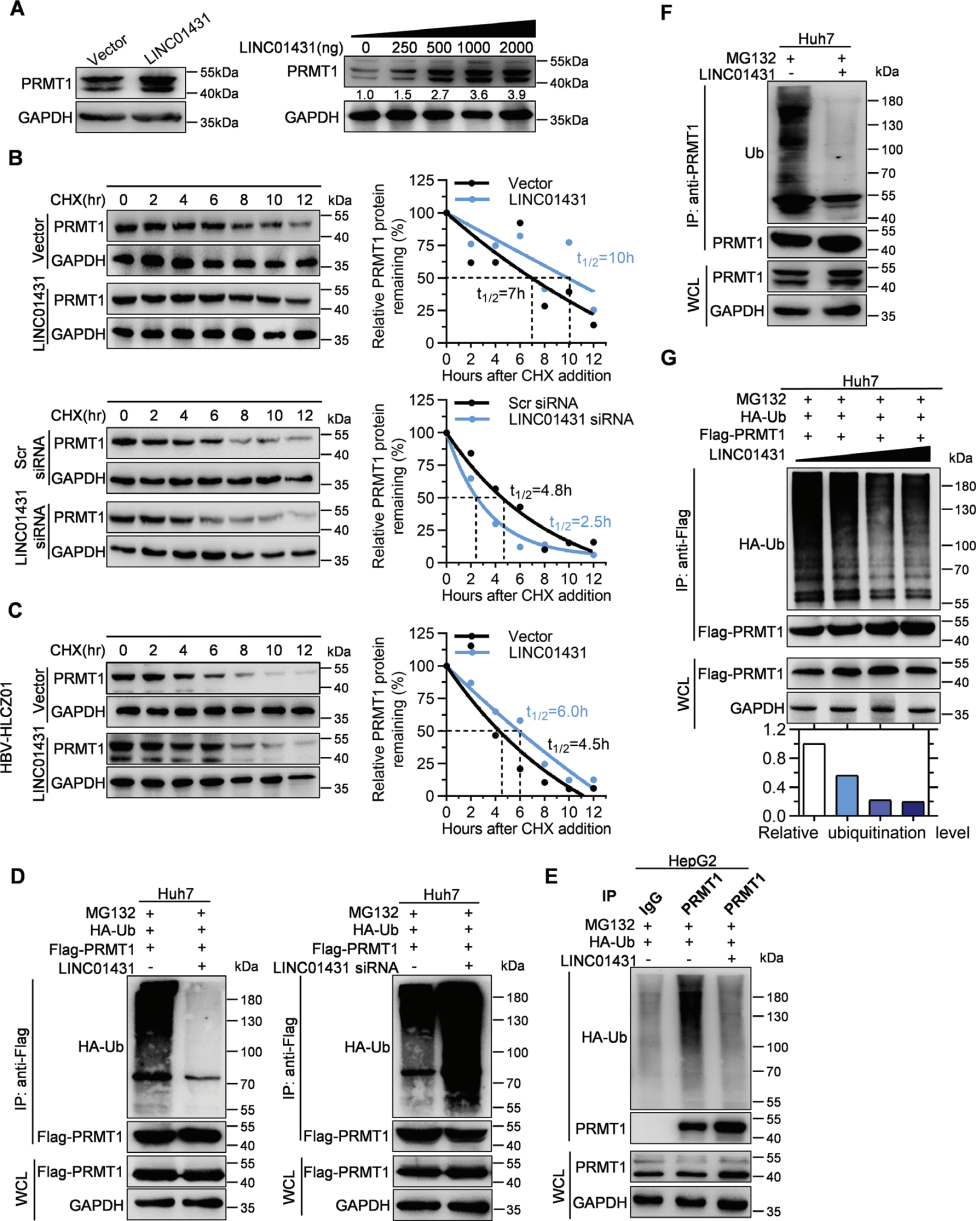
LINC01431 blocks the ubiquitination and degradation of PRMT1. A) Immunoblot analysis of PRMT1 in Huh7 cells transfected with LINC01431. B) Immunoblot analysis for the half‐life of PRMT1 in Huh7 cells transfected with LINC01431 or LINC01431 siRNA following treatment with the protein synthesis inhibitor CHX. C) Immunoblot analysis for the half‐life of PRMT1 in HBV‐infected HLCZ01 cells transfected with LINC01431 following treatment with CHX. D–G) Ubiquitination of PRMT1 in HCC cells. D) Ubiquitination of exogenous PRMT1 in Huh7 cells transfected with Flag‐PRMT1 and LINC01431 (left) or LINC01431 siRNA (right). E,F) Ubiquitination of endogenous PRMT1 in HepG2 cells (E) and Huh7 cells (F) transfected with LINC01431. G) Huh7 cells were transfected with Flag‐PRMT1 and an increased dose of LINC01431. Cells were cultured with the presence of MG132, and the ubiquitination of exogenous PRMT1 (D,G) or endogenous PRMT1 (E,F) was detected at day 3 post transfection by immunoprecipitating with anti‐Flag or anti‐PRMT1 antibodies, followed by immunoblotting with indicated antibodies. PRMT1 and GAPDH served as the loading control. CHX (400 µg mL^−1^) was used to inhibit the protein‐synthesis of PRMT1, and MG132 (20 µm) was added before protein extraction to inhibit PRMT1 degradation. The relative ubiquitination level was calculated as follows: relative ubiquitination level = band density ratio (ubiquitination band density / immunoprecipitated PRMT1 band density) (set as 1 at control group). For all experiments, representative of 3 independent experiments.

Accumulating evidence has revealed the role of lncRNAs in regulating protein stability.^[^ ^28^, ^29^ ^]^ We therefore came to evaluate whether LINC01431 could determine the fate of PRMT1 protein. Interestingly, ectopic LINC01431 expression increased PRMT1 protein abundance in Huh7 cells in a dose‐dependent manner (**Figure** **4**A). However, the levels of PRMT1 mRNA remained unchanged in HCC cells either with LINC01431 overexpression or knockdown (Figure S4A,B, Supporting Information). Consistently, following treatment with the protein synthesis inhibitor cycloheximide (CHX), LINC01431 overexpression increased the half‐life of PRMT1 protein, whereas knockdown of LINC01431 accelerated the degradation of PRMT1 in Huh7 cells (Figure 4B). Furthermore, the critical role of LINC01431 in determining the protein stability of PRMT1 was validated in HBV‐infected hepatocytes. As shown in Figure 4C, ectopic expression of LINC01431 markedly enhanced the half‐life of PRMT1 in HBV‐infected HLCZ01 cells. These results suggest that LINC01431 enhances PRMT1 protein abundance by increasing its stability.

Ubiquitination plays a crucial role in regulating the stability of multiple proteins.^[^ ^30^ ^]^ We thus came to explore whether LINC01431 is involved in modulating PRMT1 ubiquitination. As shown in Figure 4D and Figure S4C, Supporting Information, following treatment with the proteasome inhibitor MG132, the ubiquitination of exogenous PRMT1 was significantly reduced in LINC01431‐overexpressed cells, while knockdown of LINC01431 markedly increased the accumulation of ubiquitinated Flag‐PRMT1 in Huh7 cells and HEK293 cells. Concurrently, similar results were obtained from endogenous PRMT1 (Figure 4E,F), and LINC01431 reduced the ubiquitination of PRMT1 in a dose‐dependent manner (Figure 4G).

**Figure 5:**
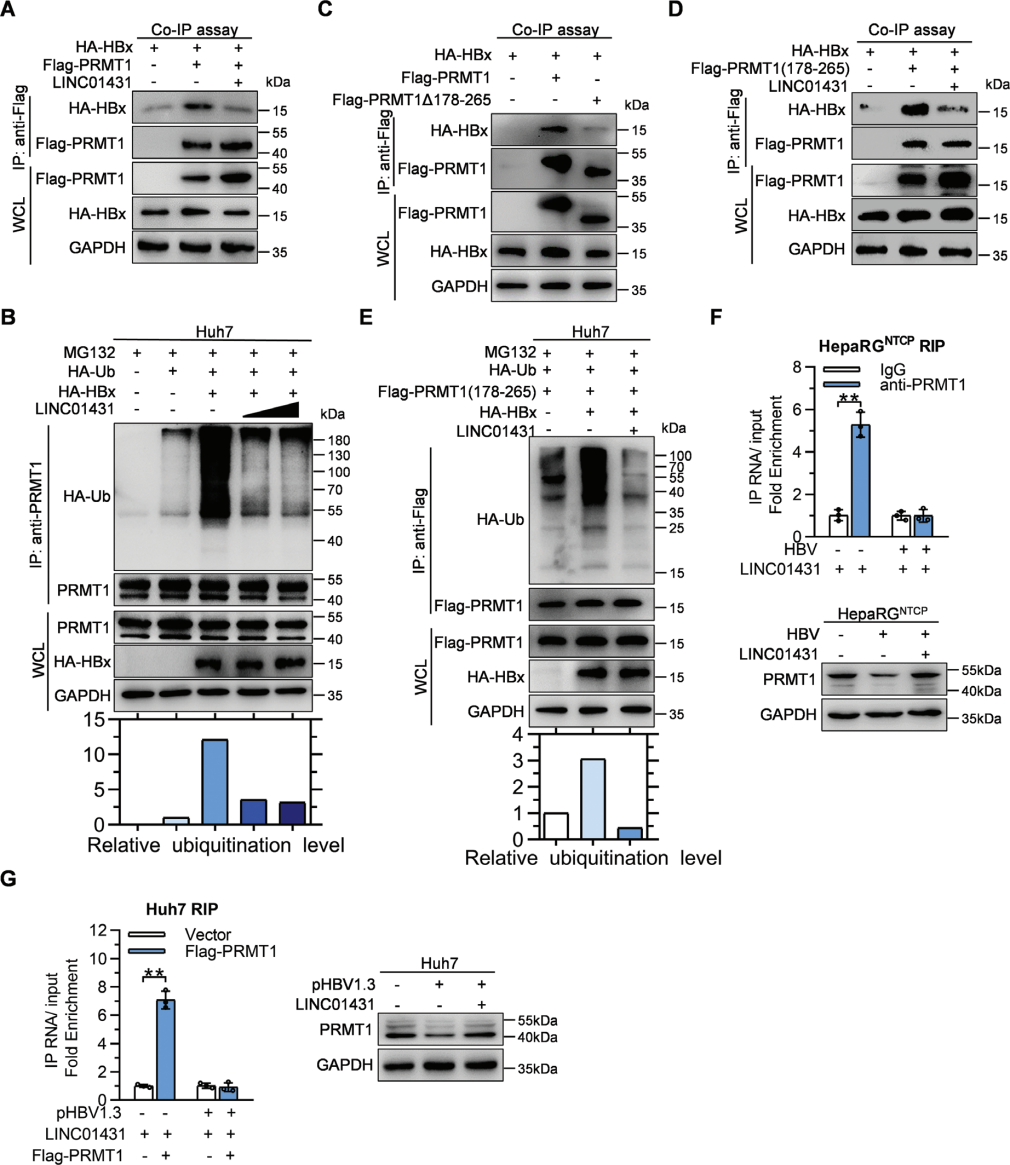
LINC01431 abolishes the HBx‐mediated ubiquitination and degradation of PRMT1. A,C,D) Co‐IP assays evaluating the interaction between PRMT1 and HBx with or without the presence of LINC01431. Huh7 cells were transfected with indicated plasmids, and the CoIP assay was performed at day 3 post transfection using anti‐Flag antibody, followed by immunoblotting with indicated antibodies. B,E) Ubiquitination assay of PRMT1 in Huh7 cells with the presence of MG132, HA‐HBx, and HA‐Ub. The ubiquitination of PRMT1 was detected by immunoprecipitation, followed by immunoblotting with indicated antibodies. F,G) Immunoblot and RIP assay evaluating the interaction of LINC01431 and PRMT1 with or without the presence of HBV. F) HepaRG^NTCP^ cells transfected with LINC01431 were cultured with or without HBV infection, the cells were harvested at 5 dpi and subjected to RIP assay (upper). HepaRG^NTCP^ cells infected with HBV were cultured with or without the presence of LINC01431, the cells were harvested at 5 dpi and subjected to immunoblot (bottom). G) Huh7 cells were transfected with LINC01431 and Flag‐PRMT1 with or without the presence of pHBV1.3, the cells were harvested at day 3 post transfection and subjected to RIP assay (left). Huh7 cells were transfected with pHBV1.3 with or without the presence of LINC01431, the cells were harvested at day 3 post transfection and subjected to immunoblot (right). For immunoblot, MG132 (20 µm) was added before protein extraction to inhibit PRMT1 degradation. RIP was performed with anti‐PRMT1 (F) or anti‐Flag (G) antibody, GAPDH served as the loading control. LINC01431 was amplified by RT‐qPCR and normalized data were shown as relative fold enrichment to the control group. For all experiments, representative of 3 independent experiments. Data information: data were presented as mean ± SD and normalized to the control group. Two‐tail unpaired Student's *t*‐tests; ^*^ *p* < 0.05; ^**^ *p* < 0.01 (F,G).

It has been reported that HBx could bind to and interfere with the DDB1‐dependent polyubiquitination of PRMT1 to promote HBV replication.^[^ ^27^, ^30^ ^]^ Thus, we next explored whether LINC01431 affects the interaction between PRMT1 and HBx. Co‐immunoprecipitation (CoIP) assays confirmed the interaction of HBx with PRMT1 in Huh7 cells, while LINC01431 overexpression significantly abolished their interaction (**Figure** **5**A). Moreover, HBx overexpression markedly increased the ubiquitination of PRMT1, which was consistent with previous reports,^[^ ^27^, ^30^ ^]^ while LINC01431 overexpression significantly reduced the HBx‐mediated ubiquitination on PRMT1 (Figure 5B). Given that the 178–265 domain of PRMT1 is required for its interaction with LINC01431, we then examined whether it is responsible for its interaction with HBx. The deletion of 178–265 domain (PRMT1∆178‐265) decreased the ability of PRMT1 to interact with HBx in Huh7 cells (Figure 5C), while the truncated PRMT1 containing only 178–265 domain (PRMT1(178‐265)) retained the ability to bind with HBx, and LINC01431 overexpression repressed these interactions (Figure 5D). Furthermore, HBx overexpression significantly increased the ubiquitination of PRMT1(178‐265) in Huh7 cells, which was markedly inhibited by ectopic LINC01431 expression (Figure 5E).

To further confirm the key findings above, HBV‐infected HepaRG^NTCP^ cells were employed. Consistently, HBV infection markedly reduced the protein level of PRMT1, while LINC01431 overexpression significantly rescued the decreased PRMT1 level in HBV‐infected HepaRG^NTCP^ cells (Figure 5F bottom). In addition, RIP assay showed that HBV infection completely revoked the interaction between LINC01431 and PRMT1 in HBV‐infected HepaRG^NTCP^ cells (Figure 5F upper). Concurrently, similar results were obtained in pHBV1.3‐transfected Huh7 cells (Figure 5G). Together, the above results suggest that LINC01431 enhances the stability of PRMT1 protein via abolishing the recruitment of HBx to PRMT1 and preventing PRMT1 degradation.

### 2.5 LINC01431 Increases the Occupancy of PRMT1 on HBV cccDNA to Repress cccDNA Transcription

**Figure 6:**
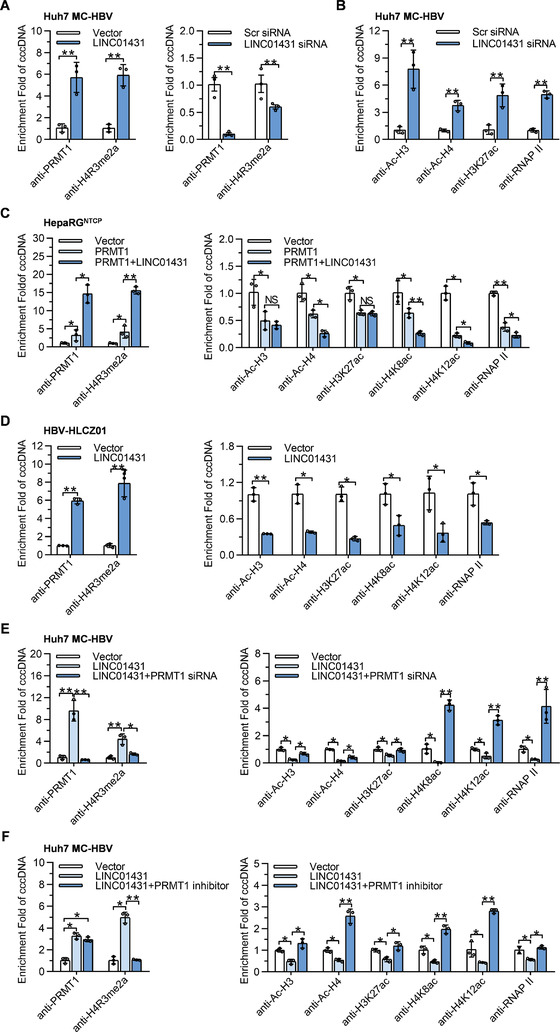
LINC01431 increases the occupancy of PRMT1 on HBV cccDNA, which promotes H4R3me2a and induces cccDNA silencing. ChIP assays were performed in MC‐HBV‐transfected Huh7 (A,B,E,F), HBV‐infected HepaRG^NTCP^ (C), and HBV‐infected HLCZ01 cells (D). A–D) ChIP assays were performed in Huh7 cells, HepaRG^NTCP^ cells, and HLCZ01 cells with LINC01431 overexpression or knockdown, and the enrichment of PRMT1 and H4R3me2a as well as epigenetic modifications were detected using indicated antibodies. E,F) Rescue assays were performed in MC‐HBV‐transfected and LINC01431 overexpressed Huh7 cells after silencing PRMT1 or in the presence of the PRMT1 inhibitor C‐7280948 (12.8 µm). The enrichment of PRMT1, H4R3me2a, and epigenetic modifications on cccDNA were measured using indicated antibodies. For all experiments, representative of 3 independent experiments. Data information: data were presented as mean ± SD and normalized to the control group. One‐way ANOVA (C,E,F). Two‐tail unpaired Student's *t*‐tests; *p < 0.05; **p < 0.01; NS, no significance (A,B,D).

PRMT1 is a type I protein arginine methyltransferase and catalyzes the asymmetric dimethylation of arginine 3 on histone H4 (H4R3me2a),^[^ ^31^, ^32^, ^33^ ^]^ which can bind to HBV cccDNA.^[^ ^27^ ^]^ Therefore, we next investigate whether LINC01431 affects the occupancy of PRMT1 on cccDNA. ChIP assays with cccDNA specific primers showed that LINC01431 overexpression significantly increased the enrichment of endogenous PRMT1 and H4R3me2a on cccDNA in Huh7 cells (**Figure** **6**A left). While knockdown of LINC01431 markedly decreased the occupancies of both PRMT1 and H4R3me2a on cccDNA (Figure 6A right and Figure S5A, Supporting Information). It has been reported that arginine‐specific methylation of histones may cooperate with other types of PTMs to remodel chromatin structure, among which histone acetylation is the major modification that functions as a transcription regulator.^[^ ^34^, ^35^ ^]^ To link H4R3me2a to histone acetylation, ChIP assay was performed, and the result confirmed the occupancies of PRMT1 and histone modifications such as H4K8ac and H4K12ac on cccDNA (Figure S5B, Supporting Information).^[^ ^36^, ^37^ ^]^ We thus speculated whether the increased enrichment of PRMT1 on cccDNA could lead to alteration of the acetylation status on cccDNA‐bound histones. Interestingly, knockdown of LINC01431 significantly increased the enrichment of Ac‐H3, Ac‐H4, H3K27ac, as well as RNA polymerase II on cccDNA in MC‐HBV‐transfected Huh7 cells (Figure 6B). Concurrently, PRMT1 overexpression greatly increased the enrichment of H4R3me2, but decreased the enrichment of Ac‐H3, Ac‐H4, H3K27ac, H4K8ac, H4K12ac, as well as RNA polymerase II on cccDNA in HBV‐infected HepaRG^NTCP^ cells and MC‐HBV‐transfected HepG2 cells (Figure 6C and Figure S5C, Supporting Information), which is consistent with the role of PRMT1 serving as an HBV restriction factor.^[^ ^27^ ^]^ In line with the role of LINC01431 in enhancing PRMT1 protein stability, LINC01431 overexpression further increased the occupancies of PRMT1 and H4R3me2a, while significantly decreased the enrichment of Ac‐H3, Ac‐H4, H4K8ac, H4K12ac, and RNA polymerase II on cccDNA in HBV‐infected HepaRG^NTCP^ and HLCZ01 cells as well as in MC‐HBV‐transfected HepG2 cells (Figure 6C,D and Figure S5C, Supporting Information). Furthermore, knockdown of PRMT1 or treatment with the PRMT1 inhibitor C‐7280948 almost completely rescued the LINC01431‐initiated enrichment of H4R3me2a and decreased enrichment of histones acetylation and RNA polymerase II on cccDNA (Figure 6E,F). To further confirm the specific effect of LINC01431‐PRMT1 complex on cccDNA minichromosome, the primers for host genomic DNA RPL30, GAPDH, and MYOD1 were employed in ChIP assay. As shown in Figure S5D,E, Supporting Information, LINC01431 and PRMT1 overexpression had little effect on the accumulation of PRMT1, RNA polymerase II, and histone modifications on these cellular DNAs. Taken together, these data suggest that LINC01431 preferentially promotes PRMT1 binding to cccDNA, further results in decreased accessibility and epigenetic silence of cccDNA and thus inhibiting HBV transcription.

### 2.6 HBx Transcriptionally Inhibits LINC01431 Expression via Manipulating ZHX2

**Figure 7:**
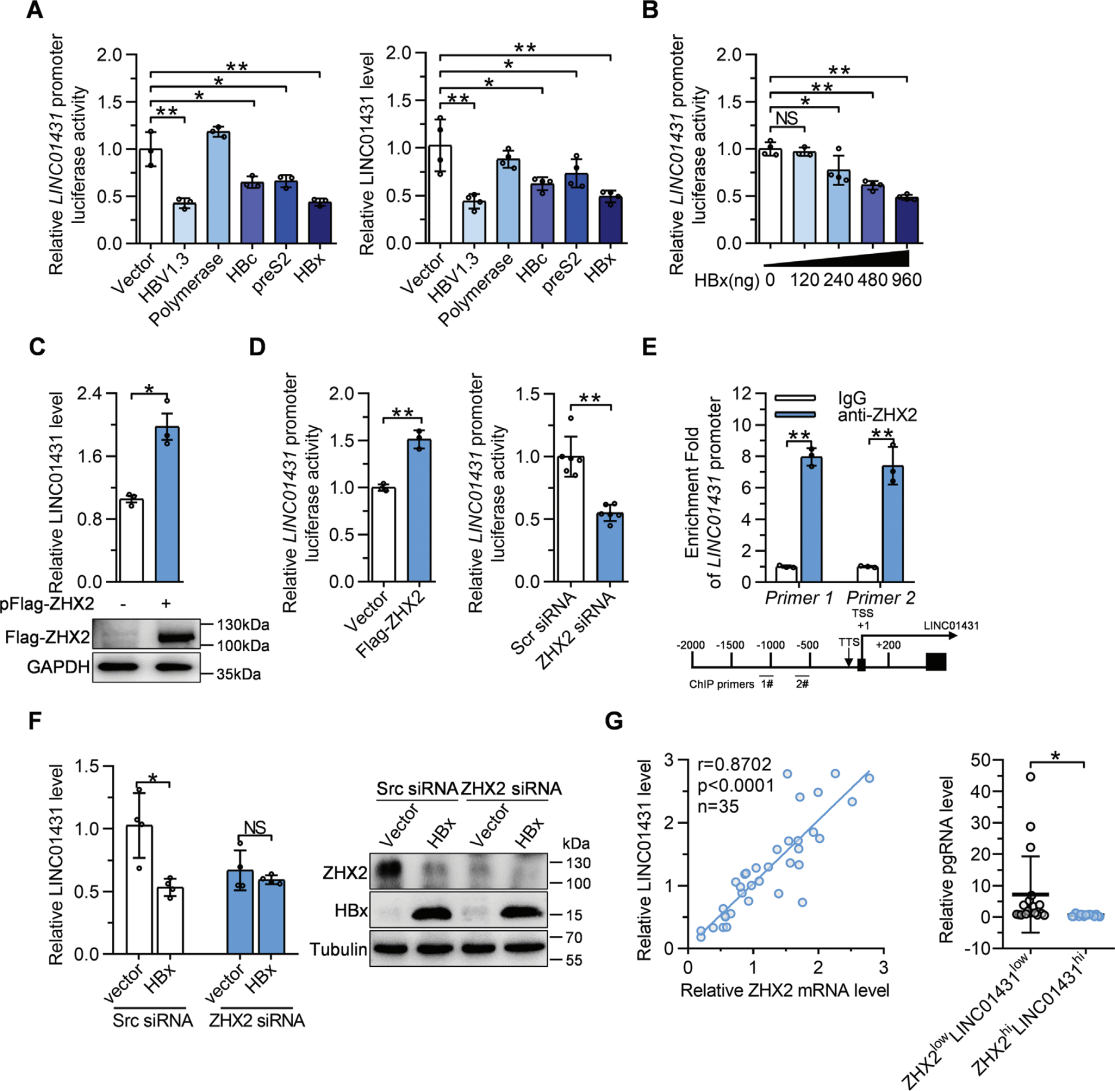
HBx transcriptionally represses LINC01431 expression via manipulating transcription factor ZHX2. A) Huh7 cells were transfected with *LINC01431* promoter and different HBV plasmids, the relative promoter activity was detected at day 2 post transfection using dual luciferase assay (left). Levels of LINC01431 were measured using RT‐qPCR (right). B) Luciferase reporter assay of *LINC01431* promoter in HBx‐transfected Huh7 cells. C) Huh7 cells were transfected with Flag‐ZHX2, LINC01431 and ZHX2 expression were detected using RT‐qPCR and immunoblotting, respectively. D) Luciferase reporter assay of *LINC01431* promoter in HepG2 cells in the presence of Flag‐ZHX2 (left) or ZHX2 siRNA (right). E) ChIP assay was performed in HepG2 cells, and the enrichment of ZHX2 on the *LINC01431* promoter was determined using the anti‐ZHX2 antibody. Normalized data were shown as relative fold enrichment to the IgG group. F) Rescue assay was performed in HBx‐transfected cells after silencing ZHX2. HepG2 cells were transfected with HA‐HBx and ZHX2 siRNAs for 3 days, LINC01431 expression was measured using RT‐qPCR, ZHX2, and HBx expression were evaluated by immunoblotting. G) Correlation analysis between LINC01431 and ZHX2 RNA in HCC para‐tumor tissues (left, *n* = 35). Patients were stratified according to the median value of ZHX2 RNA, and the levels of pgRNA were compared between ZHX2^low^LINC01431^low^ and ZHX2^hi^LINC01431^hi^ groups (right). For (A–F), representative of 3 independent experiments. Data information: data were presented as mean ± SD and normalized to the control group. Pearson's correlation coefficient (G). Two‐tail unpaired Student's *t*‐tests (A–F); *p < 0.05; **p < 0.01; NS, no significance (A–G).

It has been well known that HBV develops multiple strategies to escape from host restriction, among which HBV proteins‐mediated transcriptional regulation has attracted wide attention.^[^ ^2^, ^16^, ^38^ ^]^ As shown in Figure 1, HBV inhibited LINC01431 expression in an HBx‐dependent manner. Here, we came to determine whether HBx is the crucial viral protein that inhibits LINC01431 expression. Luciferase reporter assays and RT‐qPCR showed that three HBV encoded proteins with transactivation activity, including HBx, HBc, and preS2, inhibited the transcriptional activity of *LINC01431* promoter, among which HBx showed the predominant effect and repressed the transcriptional activity of *LINC01431* promoter in a dose‐dependent manner (**Figure** **7**A,B and Figure S6A, Supporting Information).

To further elucidate the molecular mechanism by which HBx inhibited LINC01431 transcription, the potential transcription factors‐binding sites in the promoter region of *LINC01431* were predicted using the online databases PROMO and JASPAR. The common enriched transcription factors in these databases were selected as the potential candidates, and were compared with the reported HBx‐regulated transcription factors in the literatures. Venn analysis identified NF‐YA, the binding partner of ZHX2,^[^ ^39^, ^40^ ^]^ as one of the enriched transcription factors (Figure S6B, Supporting Information). ZHX2 is a newly identified transcription factor that transcriptionally regulates the expression of several noncoding RNA including lncRNA, and is the target of HBx.^[^ ^40^, ^41^ ^]^ A previous study reported that ZHX2 inhibited HBV replication in multiple HBV systems.^[^ ^42^ ^]^ All the evidence inspired us to explore whether ZHX2 is involved in HBx‐mediated repression of LINC01431 expression. As shown in Figure 7C and Figure S6C–E, Supporting Information, ZHX2 overexpression significantly upregulated the level of LINC01431, while knockdown of ZHX2 decreased LINC01431 expression in different HCC cells. In accordance, luciferase reporter assays showed that ZHX2 overexpression significantly increased, while knockdown of ZHX2 decreased the transcriptional activity of *LINC01431* promoter in HepG2 cells (Figure 7D). Meanwhile, ChIP assays detected a significant enrichment of ZHX2 on *LINC01431* promoter (Figure 7E and Figure S6F, Supporting Information). To further confirm the involvement of ZHX2 in HBx‐mediated inhibition of LINC01431, ZHX2 specific siRNAs were transiently transfected into HBx‐overexpressed HCC cells. As expected, HBx overexpression significantly decreased the level of LINC01431, while knockdown of ZHX2 abolished the HBx‐mediated inhibition of LINC01431 (Figure 7F), implying that HBx inhibits LINC01431 expression in a ZHX2 dependent manner. In line with in vitro data, RT‐qPCR analysis of HCC para‐tumor tissues displayed a markedly positive correlation between ZHX2 and LINC01431 expression, and the levels of pgRNA in HCC patients with ZHX2^low^LINC01431^low^ were significantly higher than those in ZHX2^hi^LINC01431^hi^ patients (Figure 7G). Collectively, our data suggest that HBx transcriptionally inhibits LINC01431 expression via manipulating the transcriptional factor ZHX2.

## 3 Discussion

Chronic HBV infection greatly increases the risk of terminal liver diseases, and cccDNA plays a crucial role in viral persistence and antiviral therapy resistance.^[^ ^11^ ^]^ Formation and transcription of cccDNA minichromosome are regulated by epigenetic modifications, in which host factors and viral proteins are involved.^[^ ^43^ ^]^ Among all HBV viral proteins, HBx is one of the predominant HBV encoded proteins that exerts crucial functions in establishing and maintaining a transcriptionally active cccDNA in pleiotropic mechanisms.^[^ ^44^, ^45^ ^]^ HBx binds with DDB1 to stimulate HBV transcription by enhancing the viral mRNA levels.^[^ ^46^ ^]^ HBx transactivates HAT1 and promotes histones acetylation on cccDNA to enhance cccDNA transcription,^[^ ^2^ ^]^ HBx also interacts with lncRNA DLEU2 to promote persistent cccDNA transcription.^[^ ^12^ ^]^ Therefore, identification of host factors targeted by HBx for promoting cccDNA transcription is of great importance, and is believed to be beneficial for developing new HBV intervention strategies.^[^ ^12^ ^]^ Here, we identified a novel lncRNA named LINC01431 as the target of HBx, by which HBx regulate cccDNA epigenetic modifications and HBV transcription. Our findings represent a new regulation mechanism of cccDNA transcription and indicate a novel target for HBV intervention.

Accumulating studies have reported that lncRNAs participate in the regulation of HBV transcription by binding with epigenetic modification complexes.^[^ ^2^, ^47^ ^]^ Here, using RNA‐pull down assays coupled with mass spectrometry, we identified PRMT1, a type I protein arginine methyltransferase previously reported as the HBV negative regulator and the substrate of HBx‐DDB1 mediated E3 ligase,^[^ ^27^ ^]^ as the binding partner of LINC01431. We showed by RIP, Co‐IP, and ChIP assays that LINC01431 competitively binds to PRMT1 with HBx, leading to enhanced protein stability of PRMT1 and augmented enrichment of PRMT1 on cccDNA. However, the LINC01431‐mediated regulation of PRMT1 stability does not necessarily need HBx since LINC01431 significantly inhibited the ubiquitination of PRMT1 and enhanced PRMT1 protein abundance in HBV‐null HCC cells. Truncation analysis revealed that the regions of 1–190 or 291–500 nt of LINC01431, both of which could form flat stem‐loop structures, are required for its interaction with PRMT1, consisting with the scaffolds function of lncRNA to assemble protein modification complexes.^[^ ^29^, ^48^ ^]^ On the other hand, the 178–265 domain of PRMT1 is responsible for its interaction with LINC01431 and HBx, and LINC01431 overexpression diminishes the HBx‐induced PRMT1(178‐265) ubiquitination. These results highlight the protective function of LINC01431 on PRMT1 during HBV replication.^[^ ^27^ ^]^ LINC01431 interacts with PRMT1 and inhibits HBV transcription by modulating the epigenetic modifications on cccDNA‐bound histones in a PRMT1‐dependent manner. Our results also showed that, compared with host genomic DNA, PRMT1 more preferentially binds to HBV cccDNA, indicating the specific role of LINC01431‐PRMT1 in negative regulation of cccDNA epigenetic modifications and transcription. Therefore, targeting PRMT1, especially with the help of agonists to restore the inhibitory function of PRMT1, might provide a new therapeutic strategy for clinical HBV treatment. However, it should be noted that LINC01431 is a novel lincRNA with its functions rarely studied, we cannot rule out the possibility that LINC01431 might regulate host gene transcription by unknown mechanisms. Hence, further studies are required to exclude the potential side effects of the strategy targeting LINC01431.

Genome‐wide mapping of PTMs on cccDNA minichromosome revealed multiple epigenetic modifications directly related to HBV transcription, including histone methylation and acetylation.^[^ ^10^ ^]^ Histone methylation and acetylation are two major posttranslational modifications that may cooperate with each other and lie in the center of epigenetic regulation of both host and viral genes transcription.^[^ ^37^ ^]^ Methyltransferases and acetyltransferases are loaded on cccDNA and catalyze the epigenetic modifications on cccDNA‐bound histones and therefore have gained widespread attention due to their functions in regulating cccDNA transcription and as the potential targets for silencing cccDNA transcription, which may foster a functional cure of HBV infection.^[^ ^43^, ^49^ ^]^ Here, our data showed that the increased PRMT1 loading and H4R3me2a enrichment on cccDNA induced epigenetic silencing of cccDNA, which is consistent with previous studies confirming PRMT1 as a repressor of HBV transcription.^[^ ^33^, ^35^, ^43^ ^]^ As expected, the cccDNA‐bound histones were hypoacetylated, displaying as decreased enrichment of Ac‐H3, Ac‐H4, H3K27ac, H4K8ac, and H4K12ac on cccDNA minichromosome in PRMT1 overexpressed hepatocytes. However, it has been previously reported that methylation of H4R3 mediated by PRMT1 facilitates subsequent acetylation of H4K8 and H4K12 of nuclear hormone receptor.^[^ ^50^ ^]^ The detailed mechanisms by which LINC01431‐PRMT1 initiates the hypoacetylation of cccDNA‐bound histones need to be further investigated.

HBx, as one of the most critical viral proteins, facilitates HBV replication by multiple strategies.^[^ ^44^ ^]^ HBx enhances viral polymerase activity in calcium signaling‐dependent manners^[^ ^51^ ^]^ and promotes pgRNA encapsulation by increasing phosphorylation of the viral core protein.^[^ ^52^ ^]^ In addition, recent studies have shown that HBx binds with CRL4 E3 ubiquitin ligase to promote ubiquitination degradation of SMC5/6 complex to promote HBV replication.^[^ ^21^ ^]^ Here, our data elucidate a novel pathway by which HBx facilitates HBV to escape from host restrictions. Hence, HBx represses LINC01431 expression and subsequently disassociates the LINC01431‐PRMT1 complex, leading to enhanced HBx‐PRMT1 interaction and augmented ubiquitination degradation of PRMT1. Interestingly, ChIP assays using anti‐SMC5 antibody showed that LINC01431 overexpression did not affect SMC5 accumulation on HBV cccDNA (data not shown). Moreover, compared with the increased protein level of PRMT1, LINC01431 overexpression had little effect on the protein level of SMC5 (data not shown), suggesting that the protective function of LINC01431 is specifically limited to PRMT1. Mechanically, our data revealed that HBx represses LINC01431 expression by downregulating ZHX2 transcription. Collectively, these results suggest that targeting HBx is a novel strategy for HBV therapy.^[^ ^53^ ^]^

**Figure 8:**
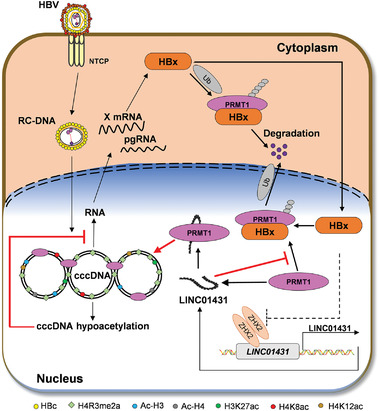
Graphical illustration of the interaction between LINC01431 and PRMT1 in repressing HBV cccDNA transcription. In this study, LINC01431 interacts with PRMT1 to block HBx‐mediated ubiquitination and degradation. As a result, LINC01431 increases the occupancy of PRMT1 on cccDNA and catalyzes the H4R3me2a, leading to cccDNA‐bound histones hypoacetylation and cccDNA silencing. In turn, HBx transcriptionally represses LINC01431 expression by targeting ZHX2 to evade host restriction. Collectively, our study presents a novel HBx‐LINC01431‐PRMT1 feedback loop responsible for regulating HBV transcription, which might provide novelty strategies for HBV intervention.

In summary, the present study for the first‐time linked LINC01431 to cccDNA epigenetic modulation and mapped the precise mechanism by which HBx‐LINC01431‐PRMT1 feedback loop promotes HBV evasion, which might provide a novel strategy for HBV treatment (**Figure** **8**).

## 4 Experimental Section

### Cell Lines and Primary Tumor Samples

HepaRG^NTCP^ cells were constructed and maintained as described.^[^ ^54^ ^]^ Briefly, Williams' Medium E (Gibco, Carlsbad, California, USA) supplemented with 10% fetal bovine serum (FBS, Gibco, Carlsbad, California, USA), 1% PS (penicillin and streptomycin, Solarbio, Beijing, China), 0.023 IE/mL insulin, 5 µg mL^−1^ hydrocortisone, and 80 µg mL^−1^ gentamicin was used. HepAD38 cells were cultured in Dulbecco's modified Eagle medium (DMEM, Gibco) supplied with 10% FBS and 1% PS. HCC cell line HLCZ01 was a friendly gift from Haizhen Zhu^[^ ^18^ ^]^ and cultured in DMEM/F12K medium supplied with 10% FBS. HCC cell line Huh7 and human embryonic kidney cell line 293 were cultured in DMEM medium supplied with 10% FBS and 1% PS. Human hepatoma cell lines HepG2 were cultured in minimum essential medium (MEM, Gibco) with 1 mmol L^−1^ sodium pyruvate. HepG2.2.15 cells were cultured in minimum essential medium (MEM) with 380 µg mL^−1^ G418 (Gibco). Huh7^NTCP^ and HepG2^NTCP^ cells were constructed as previously described.^[^ ^23^, ^55^ ^]^ Briefly, the pcDNA3‐NTCP plasmid was constructed by cloning full‐length human NTCP coding sequences into the pcDNA3.0 expression vector. Then, the plasmid was linearized and transfected into Huh7 and HepG2 cells, after three weeks culture in the presence of G418, the cell lines stably expressing the highest NTCP levels (referred to Huh7^NTCP^ and HepG2^NTCP^, respectively) were obtained (see in detail in the Supporting Experimental Section). All cells except HLCZ01 were purchased from the Shanghai Institute of Cell Biology (Chinese Academy of Sciences, Shanghai, China) and cultured in 37 °C and 5% CO_2_. Forty‐one HCC patient para‐tumor tissues were obtained from patients admitted to Qilu Hospital, Shandong University. The tissue specimens were snap‐frozen in liquid nitrogen and stored at −80 °C for RNA or protein extraction. The use of resected human samples was approved by the Medical Ethical Committee of School of Basic Medical Sciences of Shandong University (Approval Number: ECSBMSSDU2019‐1‐012), and all participants were informed and provided written informed consent. A summary of the clinical information for the 41 patients is available in Table S1, Supporting Information.

### Animal Studies

Six to eight‐week‐old male C57BL/6J mice purchased from Charles River Laboratories (Beijing, China) were fed under specific pathogen‐free conditions in laboratory animal center of School of Basic Medical Science of Shandong University. HBV carrier mice were prepared by hydrodynamic injection (HDI) of pAAV‐HBV1.2 plasmid (6 µg/mouse). To evaluate the role of LINC01431 in HBV replication, SB100 (0.8 µg/mouse) and LINC01431‐PT3EF1*α* (20 µg/mouse) were injected into mice at the same time of HDI, lncRNA GAS5‐PT3EF1*α* (20 µg/mouse) as the negative control. Hydrodynamic injection was performed as described previously.^[^ ^56^ ^]^ The total volume plasmids were diluted in a volume of normal saline equivalent to 8% of the mouse body weight and delivered into the tail veins within 5–8 s. After 3 days incubation, mouse was received Entecavir (0.1 mg kg^−1^ B. W., HY‐13623, MCE) by oral gavage once daily as the positive control. All animal experimental procedures were performed in accordance with the Shandong University guidelines for animal experiments and approved by Ethics Committee of School of Basic Medical Sciences, Shandong University (Approval Number: ECSBMSSDU2019‐2‐031).

### LncRNA Sequencing and Data Analysis

Total RNA from HLCZ01 and HBV‐infected HLCZ01 cells were isolated and subjected to lncRNA sequencing by Annoroad Gene Technology Co., Ltd (Beijing, China). Briefly, 3 µg RNA per sample was used as the initial material for the RNA sample preparations. Ribosomal RNA was removed using the Epicentre Ribo‐Zero Gold Kits (Epicentre, USA). Subsequently, the sequencing libraries were generated following manufacturer recommendations with varied index label by NEBNext Ultra Directional RNA Library Prep Kit for Illumina (NEB, Ispawich, USA). RNA concentration of the library was measured using Qubit RNA Assay Kit in Qubit 2.0 to quantify and then dilute to 1 ng µL^−1^. Insert size was assessed using the Agilent Bioanalyzer 2100 system (Agilent Technologies, CA, USA) and qualified insert size was accurately quantified using Taqman fluorescence probe of AB Step One Plus Real‐Time PCR system. Clustering was generated by cBot cluster generation system and then was sequenced on an Illumina platform. Sequencing raw reads were preprocessed by filtering out sequencing adapters, short‐fragment reads and other low‐quality reads. The authors used HISAT2 to map the cleaned reads to the human GRCh38 reference genome. After genome mapping, Read Count for each gene was counted by HTSeq, and fragments per kilobase million mapped reads (FKPM) were calculated to represent the expression level of genes in each sample. DEseq2 package was used for differential expression analysis. Differentially expressed lncRNAs between the two groups were identified through fold change and adjusted *p* values (*q* value).

### Plasmids, siRNAs, and Reagents

PRMT1 and LINC01431 expressing plasmids were constructed by amplification of the CDS (coding sequence) of *PRMT1* and the cDNA of *LINC01431* using high fidelity polymerase (ThermoFisher, Carlsbad, California, USA) and cloned into pCMV‐3×Flag7.1 vector, pcDNA3.1 vector, pUltra‐puro, and PT3EF1*α* vector, respectively. The truncations of LINC01431 and PRMT1 were generated by KOD‐Plus‐Mutagenesis Kit (TOYOBO, Japan) as previously described.^[^ ^27^ ^]^ The pGL3‐LINC01431 promoter plasmid was constructed by cloning the predicted LINC01431 promoter (23 355 994:23 358 194, GRCh38.p13, Ensembl) into pGL3‐Basic vector (Promega, Madison, USA). The HBx, ubiquitin and ZHX2 expressing plasmids were generated in the authors’ previous studies.^[^ ^41^, ^57^ ^]^ The promoter regions of HBV BCP, SPI, SPII, and XP were constructed in their previous studies.^[^ ^42^ ^]^ And the pHBV1.3 expressing plasmid was purchased from Addgene (USA). The plasmid HBV1.1 was friendly gifted from Youhua Xie (Fudan University),^[^ ^55^, ^58^ ^]^ and the HBx mutant plasmid HBV1.1ΔHBx was constructed through inducing termination codon UGA mutation to silence initiation codon ATG of HBx gene using KOD‐Plus‐Mutagenesis Kit (TOYOBO, Japan). All constructs were verified by sequencing. The siRNAs specific for LINC01431, PRMT1 and ZHX2 were designed and synthesized from GenePharma Inc. (Shanghai, China). Streptavidin T1 magnetic beads were purchased from Invitrogen (Carlsbad, California, USA). The Fast Silver Stain Kit and Enhanced BCA Protein Assay Kit were purchased from Beytime (Shanghai, China). The proteasome inhibitor MG132 and PRMT1 specific inhibitor C‐7280948 were purchased from Selleck (Shanghai, China).

### HBV Northern Blot and Southern Blot

HepaRG^NTCP^ cells were seeded in six‐well plates and inoculated with lentivirus pUltra‐LINC01431 or control lentivirus pUltra‐Vector in the presence of 8 µg mL^−1^ polybrene (Sigma‐Aldrich, St. Louis, MO, USA) for 24 h. After sufficient washing with prewarmed PBS, the cells were cultured with a medium containing 1.8% DMSO (Sigma‐Aldrich, St. Louis, MO, USA) for 48 h. Then, the cells were infected with 100 HBV genomes per cell at the presence of 5% polyethylene glycol (PEG)‐8000 (Sigma‐Aldrich, St. Louis, MO, USA) for 24 h, and harvested at 7 days post infection. The Random Primer DNA Labeling Kit Ver. 2 (Takara Bio, Japan) and NorthernMax Kit (Invitrogen, USA) were used in the experiments. For Northern blot, total cellular RNA was extracted by TRIzol reagent (Invitrogen) according to the manufacturer's instructions. Total RNA (30 µg) was used for the Northern blot assay according to the manufacturer's protocols (Ambion; Invitrogen). For Southern blot, cccDNA from HBV‐replicating cells was extracted by a Hirt protein‐free DNA extraction procedure^[^ ^59^ ^]^ and subjected to Southern blot assay as described previously.^[^ ^60^ ^]^ The Hybridization signal was collected by Typhoon FLA 9500 imager (GE Healthcare Lifesciences).

### RNA FISH and Immunofluorescence Staining Assay

RNA FISH assay was performed using the Ribo Fluorescent In Situ Hybridization Kit (RIBOBIO, Guangdong, China) according to the manual. Briefly, the cells were fixed with 4% paraformaldehyde at room temperature for 30 min. After sufficient washing, the cells were permeabilized with 0.5% Triton X‐100, followed by incubating with Cy3‐labelled probes for 18S, U6, and LINC01431 at 37 ℃ overnight, respectively. After sufficient hybridization, the cells were washed 4 times with indicated washing buffer, then the nucleus was stained with DAPI (4′,6‐diamidino‐2‐phenylindole). The staining results were acquired using a Laser confocal microscope with Airyscan 780 (ZEISS, Germany). The Immunofluorescence assay was performed as previously described.^[^ ^42^ ^]^ Briefly, the cells seeded on the slides were fixed with 4% paraformaldehyde at room temperature for 10 min, and then permeabilized with 1% NP‐40 at room temperature for 5 min. After blocking with 5% bovine serum albumin (Solarbio, Beijing, China), the cells were incubated with anti‐PRMT1 polyclonal antibody at 37 ℃ for 2 h, followed by incubating with FITC‐conjugated affinipure Goat anti‐Rabbit IgG (SA00003‐2, Proteintech, USA). After washing with 0.5% PBST, the nucleus was stained with DAPI. Images were captured as described above.

### Immunohistochemistry Analysis

Paraffin‐embedded mice liver samples were sectioned at 5‐mm thickness. Antigen retrieval was performed using a microwave oven for 20 min in citrate buffer. Samples were incubated with antibodies specific for HBc (1:200, B0586, Dako, Copenhagen, Denmark)) at 37 °C for 2 h. HRP‐conjugated secondary antibodies were incubated for 1 h at room temperature. After sufficient washing, the images were obtained using microscopy (Olympus Corporation, Japan).

### Subcellular Fractionation

Cytoplasmic and nuclear fractions of HepG2 cells were prepared and separated according to the manual of the Nuclear/Cytoplasmic Isolation kit (ThermoFisher, USA) as previously described.^[^ ^28^ ^]^ 18S was used as the endogenous cytoplasmic control and U6 small nuclear RNA was used as the endogenous nuclear control. The relative distribution of LINC01431 was detected by RT‐qPCR.

### Dual Luciferase Reporter Assay

The HCC cells were seeded overnight, then co‐transfected with indicated plasmids. Approximately 48 h after transfection, the cells were lysed and subjected to luciferase activity analysis using Dual‐Luciferase Reporter Assay System (Promega, Madison, USA).

### RNA‐Pull Down Assay and Mass Spectrometry Data Analysis

The sense and antisense transcripts of LINC01431 were transcribed and biotinylated using the Ribo RNAmax‐T7 Kit (RIBOBIO, Guangdong, China) according to the manual, and RNA‐pull down assay was performed as previously described.^[^ ^61^ ^]^ Briefly, biotinylated RNA was pretreated with RNA structure buffer, then added into the precleared cell lysate and rotated at room temperature for 3 h. Then streptavidin‐coupled beads (Invitrogen, USA) were added to the reaction and rotated at room temperature for 2 h. After sufficient washing, the proteins were dissolved in 1×SDS loading Buffer (Beytime, China) and separated by SDS‐PAGE, followed by silver staining. The specific band was excised and subjected to proteomics screening by PTM BIO (Hangzhou, China), and the retrieved protein was detected by immunoblotting. To identify potential LINC01431‐interacted proteins, the number of unique peptides of detected proteins should be equal or greater than 2 (Cut‐off = unique peptide ≥ 2). Then, through the Venn analysis, the LINC01431 antisense transcript‐interacted proteins were excluded in LINC01431‐sense group, and the nuclear‐localized proteins with the molecular weight ≈40 kDa were selected as the potential candidates for further analysis. Finally, the top‐scored proteins were selected as the potential targets and verified using RNA pull‐down and RIP assays.

### RIP Assay

The cells were harvested and lysed in cell lysis buffer (Beytime, China) supplied with protease inhibitor and RNase inhibitor. Anti‐Flag antibody (MBL, Beijing, China) was added into the clarified lysate and hybridized on the rotator at 4 ℃ for 4 h. The ChIP‐Grade Protein G Magnetic Beads (Cell Signaling Technology, Danvers, Massachusetts, USA) were added into the reaction and hybridized at room temperature for 2 h. The precipitated RNA was washed 5 times using washing buffer (150 mm KCl, 25 mmTris pH 7.4, 5 mm EDTA, 0.5 mm DTT, 0.5% NP40, and 0.1% DEPC). After sufficient washing, the retrieved RNA was isolated using TRNzol (TIANGEN, Beijing, China), then subjected to RT‐qPCR analysis.

### ChIP Assay

Chromatin immunoprecipitation (ChIP) assays were performed using the EZ‐Magna ChIP Chromatin Immunoprecipitation Kit (Millipore, Darmstadt, Germany) according to the manufacturer's instructions. Briefly, the HCC cells were fixed with 1% formaldehyde at room temperature, then suspended by adding glycine and incubating at room temperature for 5 min. Then the cells were scratched and collected into the centrifuge tube, followed by nuclear purification. The isolated cross‐linked nuclei were lysed in nuclear lysis buffer and sheared by sonication to generate 500–1000 bp DNA fragments. After centrifugation, the supernatant was diluted in dilution buffer and precleared with protein A/G magnetic beads. The protein‐DNA complexes were immunoprecipitated with indicated antibodies at 4 ℃ overnight, followed by immunoprecipitating with protein A/G magnetic beads at 4 ℃ for 2 h. The precipitated DNA was washed with indicated washing buffer, followed by digested with proteinase K to release DNA fragments. The retrieved DNA was used for both conventional PCR and RT‐qPCR with specific primers. And the results were presented as relative fold enrichment compared to the control group.

### Statistical Analysis

All statistical analyses were performed using Prism software package version 8 (GraphPad Software, San Diego, CA, USA). Unpaired Student's *t*‐test and One‐way ANOVA were applied to compare the statistical significance of differences between groups. All data were presented as mean ± standard deviation (SD) of one representative experiment. Correlation analysis between LINC01431 and HBV pgRNA, and between ZHX2 and LINC01431 were analyzed using Pearson's correlation coefficient. All statistical tests were two‐tail; *p* value of less than 0.05 was considered statistically significant, ^*^ *p* < 0.05; ^**^ *p* < 0.01, NS: no significance.

## Conflict of Interest

The authors declare no conflict of interest.

## Author Contributions

Y.S. and Y.T. are the co‐first authors. Y.S., Z.W., and C.M. conceived the project and wrote, reviewed, and edited the manuscript. Y.S. performed most of the experiments, with contributions from Y.T., L.W., Z.Z., C.C., and Y.W. X.S. and J.L. collected clinical samples. Y.S., X.Z., and P.X. collected and analyzed data. Y.S., Y.T., N.L., L.G., X.L., Z.W., Y.X., and C.M. revised the paper. All authors read and approved the final manuscript.

## Supporting information

Supporting Information

## Data Availability

The data that support the findings of this study are available from the corresponding author upon reasonable request.
