# Supplementary material for: LINC01431 Promotes Histone H4R3 Methylation to Impede HBV Covalently Closed Circular DNA Transcription by Stabilizing PRMT1
Source: Adv Sci (Weinh). 2022 Apr 10;9(16):2103135. doi: 10.1002/advs.202103135 (PMC9165498; doi:10.1002/advs.202103135)
Supplement: Supplementary file 1 — Supporting Information [file ADVS-9-2103135-s001.pdf]

ADVANCED SCIENCE
Open Access

Supporting Information

for *Adv. Sci.*, DOI 10.1002/advs.202103135

LINC01431 Promotes Histone H4R3 Methylation to Impede HBV Covalently Closed Circular DNA Transcription by Stabilizing PRMT1

Yang Sun, Yan Teng, Liyuan Wang, Zhaoying Zhang, ChaoJia Chen, Yingchun Wang, Xiaodong Zhang, Peng Xiang, Xiaojia Song, Jinghui Lu, Nailin Li, Lifen Gao, Xiaohong Liang, Yuchen Xia, Zhuanchang Wu* and Chunhong Ma**

Supporting Information

LINC01431 Promotes Histone H4R3 Methylation to Impede HBV covalently closed circular DNA Transcription by Stabilizing PRMT1

Yang Sun, Yan Teng, Liyuan Wang, Zhaoying Zhang, ChaoJia Chen, Yingchun Wang, Xiaodong Zhang, Peng Xiang, Xiaojia Song, Jinghui Lu, Nailin Li, Lifen Gao, Xiaohong Liang, Yuchen Xia*, Zhuanchang Wu*, Chunhong Ma*

Dr. Y. Sun, Dr. L. Wang, Dr. Z. Zhang, Dr. C. Chen, Dr. Y. Wang, Dr. X. Zhang, Dr. P. Xiang, Dr. X. Song, Prof. L. Gao, Prof. X. Liang, Prof. Z. Wu, Prof. C. Ma
Key Laboratory for Experimental Teratology of Ministry of Education and Department of Immunology, School of Basic Medical Sciences, Cheeloo Medical College, Shandong University, Jinan, 250012, Shandong, China
Email: zhuanchangwu@sdu.edu.cn
Email: machunhong@sdu.edu.cn

Prof. J. Lu
Department of Hepatobiliary Surgery, Qilu hospital of Shandong University, Jinan, 250012, Shandong, China

Prof. N. Li
Karolinska Institute, Department of Medicine-Solna, Clinical Pharmacology Group, 17176 Stockholm, Sweden

Dr. Y. Teng, Prof. Y. Xia
State Key Laboratory of Virology and Hubei Province Key Laboratory of Allergy and Immunology, Institute of Medical Virology, School of Basic Medical Sciences, Wuhan University, Wuhan, 430072, Hubei, China
Email: yuchenxia@whu.edu.cn

Y. Sun and Y. Teng are the co-first authors.

Supporting Experimental Section

Establishment of MC-HBV: Recombinant cccDNA, named Minicircular HBV (MC-HBV) was constructed by combining minicircle DNA technology and HBV Cre/prcccDNA model. Briefly, HBV 1.0 copy genome containing chimeric intron amplified from prcccDNA was subcloned into minimized minicircle vector retaining attB/attP sites using In-Fusion HD Cloning Kit (Clontech, USA) to form parent plasmid pmini-HBV-intron (PP).[1,2] PP was then transformed into ZYCY10P3S2T strain and induced with 0.2 mg/ml arabinose (Sigma-Aldrich, St. Louis, MO, USA) at 30°C pH 7.0 to generate MC-HBV. The plasmids were transfected into Huh7 cells to confirm the expression efficiency, HBV replication parameters were detected and compared between these groups.

Construction of NTCP-stably expressing cell lines: The Huh7^{NTCP} and HepG2^{NTCP} cells were constructed as previously described.[3,4] We transfected Huh7 and HepG2 cells with a pcDNA-hNTCP expression plasmid containing a full-length human NTCP coding sequence, which was pre-linearized using restriction enzyme PvuI without breaking the integrity of NTCP and G418-resistance gene expression cassettes prior to transfection to increase the efficiency of stable clone generation and target gene expression.[5] Then the NTCP-expressed single cell clones were selected and expanded with the presence of G418 for three weeks and the expression levels of NTCP in different clones were detected using RT-qPCR and immunofluorescence, the clones stably expressing the highest level of NTCP were termed as Huh7^{NTCP} and HepG2^{NTCP} cells and used for subsequent HBV infection experiments. The HepaRG^{NTCP} cells were constructed as previously described.[6]

Western blot and antibodies: Total cell proteins were extracted using Cell Lysis Buffer (Beytime, Shanghai, China) supplemented with protease inhibitor (Cell Signal Technology, Danvers, Massachusetts, USA). After sufficient centrifugation, the proteins were separated by SDS-PAGE, and followed by immunoblotting with indicated antibodies. Anti-AcH3 monoclonal antibody (06-599) and anti-AcH4

monoclonal antibody (06-598) were purchased from Millipore (Sigma-Aldrich, St. Louis, MO, USA). Anti-HBx monoclonal antibody (ab2741), anti-PRMT1 polyclonal antibody (ab3768), anti-H4K8ac polyclonal antibody (ab15823) and anti-H4K12ac polyclonal antibody (ab46983) were purchased from Abcam (Cambridge, MA, USA). Anti-H4R3me2a polyclonal antibody (A2376) was purchased from ABclonal (Wuhan, Hubei, China). Anti-HRP-conjugated-Flag monoclonal antibody (M185-6) and anti-HRP-conjugated-HA monoclonal antibody (M180-7) were purchased from MBL (Beijing, China). Anti-glyceraldehyde 3-phosphate dehydrogenase (GAPDH) monoclonal antibody (66004-I-Ig), anti-β-actin monoclonal antibody (66009-1-Ig), anti-Tubulin monoclonal antibody (11224-1-AP), anti-mouse HRP-immunoglobulin G (IgG) secondary antibody and anti-rabbit HRP-immunoglobulin G (IgG) secondary antibody were purchased from Proteintech (Rosemount, Illinois, USA).

RNA extraction and quantitative reverse transcription PCR (RT-qPCR): Total RNA from cells or tissue samples were extracted using TRNzol reagent (TIANGEN, Beijing, China). RNA was treated with DNase I to remove the genomic DNA and plasmids DNA, followed by reversely transcribed into cDNAs using Thermo Scientific RevertAid First Strand cDNA Synthesis Kit (ThermoFisher, USA). Conventional PCR was performed using BioRad Thermal Cycler with specific primers, and quantitative real-time PCR was performed using SYBR Green Master kit (TIANGEN, Beijing, China) in the BioRad CFX96 Real-Time system. The sequences of primers were presented in Table 2, and the expression levels of genes were presented as values normalized against *GAPDH* transcripts.

Immunoprecipitation assay: The overexpression plasmids or siRNA were transiently transfected into HCC cells. At indicated time post-transfection, the cells were harvested and lysed in Cell Lysis Buffer containing protease inhibitors on ice for 10 minutes, followed by centrifuging at 4℃, 12,000×g for 15 minutes. The supernatants were collected and incubated with anti-Flag or anti-HA Protein G Dynabeads (MBL, China) at 4°C with gentle rotation overnight. After sufficient washing, the retrieved

proteins were dissolved in 1×SDS loading buffer (Beytime, China), then separated by SDS-PAGE, followed by immunoblotting with indicated antibodies. PRMT1, HBx and GAPDH served as loading control.

Transfection and infection: Cells were transfected with overexpression plasmids or siRNA using Lipofectamine 2000 reagent according to the instructions (Invitrogen, USA). The culture medium was refreshed after 4-6 hours post transfection, and the cells were harvested at indicated time and subjected to following experiment. $Huh7^{NTCP}$, $HepG2^{NTCP}$, $HepaRG^{NTCP}$ and HLCZ01 cells were infected with concentrated HBV at 400 GEq/cell in the presence of 4% polyethylene glycol (PEG)-8000 (Sigma-Aldrich, USA) for 6 hours, and then rinsed three times with phosphate-buffered saline (PBS) and cultured in the DMEM with 10% FBS for 4-7 days, followed by indicated experiments.

Quantitation of HBV replication: The secreted HBsAg and HBeAg concentration in the cell culture medium were quantitated using commercially available HBsAg, HBeAg ELISA kits (InTec Inc., Xiamen, China). Cell culture supernatants were collected to measure the secreted core particle HBV DNA using commercial Sansure HBV DNA quantitation detection kits (Sansure Biotech, China). Intracellular HBV cccDNA was prepared from cultured cells as following. Briefly, intercellular DNA was extracted using TIANamp Genomic DNA kit (TIANGEN, China), then treated with plasmid-safe DNase I (ThermoFisher, USA) to clear the HBV rcDNA and integrated linear genomic DNA. The remaining HBV cccDNA was detected by RT-qPCR with specific primers.[7]

Protein and RNA stability assay: The cells were transfected with indicated plasmids or siRNA for 24 hours, and then treated with actinomycin D (ActD, 5 μg/mL, MCE, Shanghai, China) or cycloheximide (CHX, 400 μg/mL, MCE, Shanghai, China) for indicated time. HBV-pgRNA stability in the ActD-treated group was analyzed by RT-qPCR. The half-life of PRMT1 protein in the CHX-treated group was observed by immunoblotting.

ChIP Assay: ChIP assays were performed using the EZ-Magna ChIP™ Chromatin Immunoprecipitation Kit (Millipore, Darmstadt, Germany) according to the manufacturer's instructions. Briefly, the cells were fixed with 1% formaldehyde at room temperature for 10 minutes, then suspended by adding glycine and incubating at room temperature for 5 minutes. Then the cells were scratched and collected into the centrifuge tube, followed by nuclear purification. The isolated cross-linked nuclei were lysed in nuclear lysis buffer and sheared by sonication to generate 500-1000 bp DNA fragments. After centrifugation, the supernatant was diluted in dilution buffer and precleared with protein A/G magnetic beads. The protein-DNA complexes were immunoprecipitated with indicated antibodies at 4℃ overnight, followed by immunoprecipitating with protein A/G magnetic beads at 4℃ for 2 hours. The precipitated DNA was washed with indicated washing buffer, followed by digested with proteinase K to release DNA fragments. The retrieved DNA fragments were used for both conventional PCR and RT-qPCR with specific primers. And the results were presented as relative fold enrichment compared to the control group. Strategies for quantification and calculation were as described. Briefly, we retained 1% of total sonicated sample as Input before immunoprecipitating with indicated antibodies. DNA from ChIP group and Input group were detected by cccDNA-specific qPCR. Data were analyzed as following: $\Delta CT(Target)=CT^{ChIP(Target)}-[CT^{Input(Target)}-\log_2 100]$; the relative fold enrichment of targets on cccDNA was calculated as $2^{control\Delta CT(Target)-experimental\Delta CT(Target)}$.

References

[1] Li, F., Cheng, L., Murphy, C. M., Reszka-Blanco, N. J., Wu, Y., Chi, L., Hu, J. & Su, L., *Sci Rep* **2016**, 6, 36483.

[2] Yan, Z., Zeng, J., Yu, Y., Xiang, K., Hu, H., Zhou, X., Gu, L., Wang, L., Zhao, J., Young, J. A. T. & Gao, L., *J Hepatol* **2017**, 66, 1149.

[3] Song, X., Tan, S., Wu, Z., Xu, L., Wang, Z., Xu, Y., Wang, T., Gao, C., Gong, Y., Liang, X., Gao, L., Spear, B. T. & Ma, C., *Int J Cancer* **2018**, 143, 3120.

[4] Wu, Z., Tan, S., Xu, L., Gao, L., Zhu, H., Ma, C. & Liang, X., *Antiviral Res* **2017**, 145, 20.

[5] Stuchbury, G. & Münch, G., *Cytotechnology* **2010**, 62, 189.

[6] Ni, Y. & Urban, S., *Methods Mol Biol* **2017**, 1540, 15.

[7] Xu, L., Wu, Z., Tan, S., Wang, Z., Lin, Q., Li, X., Song, X., Liu, Y., Song, Y., Zhang, J., Peng, J., Gao, L., Gong, Y., Liang, X., Zuo, X. & Ma, C., *Antiviral Res* **2018**, 153, 114.

Supporting tables

Supporting Table 1 Clinical characteristics of enrolled subjects

Characteristics	No. of patients
Age (year)[a]	
≤55	16
>55	25
Gender	
Male	33
Female	8
HBsAg	
Positive	35
Negative	6
Differentiation	
I-II	31
III-IV	10

\[a]mean±SD

Supporting Table 2 Primers and siRNA in this study

Gene and primer	Primer sequence (5' to 3')
LINC01431-fwd	GAAACCAACAGACCTTGCTGTC
LINC01431-rev	GCTTTCTGTGTTTCACCGTCTC
pgRNA -fwd	CTCAATCTCGGGAATCTCAATGT
pgRNA-rev	AGGATAGAACCTAGCAGGCATAAT
cccDNA-PSD-fwd	TTCTCATCTGCCGGACCG
cccDNA-PSD-rev	CACAGCTTGGAGGCTTGAAC
lncRNA GAS5-fwd	AGGCATTAGACAGAAAGCTGGAA
lncRNA GAS5-rev	ATGTCCTTACCCAAGCAAGTCAT
RPL30-ChIP-fwd	CAAGGCAAAGCGAAATTGGT
RPL30-ChIP-rev	GCCCGTTCAGTCTCTTCGATT
GAPDH-ChIP-fwd	TCGACAGTCAGCCGCATCT
GAPDH-ChIP-rev	CTAGCCTCCCGGGTTTCTCT

MYOD1-ChIP-fwd	CCGCCTGAGCAAAGTAAATGA
MYOD1-ChIP-rev	GGCAACCGCTGGTTTGG
PRMT1-fwd	CCTCTTGAAGAAGTGTCCTGTGG
PRMT1-rev	CAAAGTGTGCGTAGGAGTCAAAG
ZHX2-fwd	GGTTCGGACATCACAAGTAGTAG
ZHX2-rev	GGTGTGCCGATTCCTTTCTCT
GAPDH-fwd	GGAGTCCACTGGCGTCTTCAC
GAPDH-rev	GAGGCATTGCTGATGATCTTGAGG
18S-fwd	CGGCTACCACATCCAAGGAA
18S-rev	GCTGGAATTACCGCGGCT
CTD-2587M2.1-fwd	CTTCAGTAGTTGGACTCGGTGAA
CTD-2587M2.1-rev	TTCCTAGAACCTTTGACCTTCGG
RP11-297L17.2-fwd	CTTAGAAGTCTTCCAGCCACCTT
RP11-297L17.2-rev	AGCAACACTCTGCTCAGTAACTC
RP11-159D12.2-fwd	TGCTTCTGCTACTGATCCAAACT
RP11-159D12.2-rev	CAGTGTGTTTCCTTTCCATCCAC
RP11-408B11.2-fwd	TCTGGAGGGGAGTTTCATCTTTG
RP11-408B11.2-rev	AGGAAGTTTCTGGAGGACATAGC
RP11-397A16.1-fwd	TTGGGTTGAAGACGAACAGAAGA
RP11-397A16.1-rev	CCAGATGTCTATCTCCAGCTTCC
RP1-27K12.2-fwd	GGAACACAGCATCTTCTGCAAAT
RP1-27K12.2-rev	AGCAGAGCCAGTATTTGATTCCA
RP11-622C24.2-fwd	GGGAAGCGCTCAGAAAGTATCAT
RP11-622C24.2-rev	CCCGAGCAGAGAGATTCATTACA
RP11-347I19.7-fwd	TGCAACAAAGGAAAAACCCACTT
RP11-347I19.7-rev	AGACGCTGATCTCAAAACATCCT
RP11-714M23.2-fwd	GCAAATCTGAAAAGTGACGGTGA
RP11-714M23.2-rev	CCCACTAATTCTCTTTGGCCTCT

AC017060.1-fwd	GCAGCAGATGGTCCTGTATCTAA
AC017060.1-rev	TCCCAGATTCTCTTGCCTGTTTT
**siRNA**	**siRNA sequence (5’ to 3’)**
LINC01431-siRNA	S1: CCUUUCCUCAACGUUACCATT
	S2: GCUUGCAACAGACCACAUUTT
	S3: GCACUGCGUGCUGAUUAUATT
PRMT1-siRNA	S1:CGUGUAUGGCUUCGACAUGTT
	S2: UCAAAGAUGUGGCCAUUAATT
	S3: CGUCAAAGCCAACAAGUUATT
ZHX2-siRNA	S1: GCAGAACUGGAUCGGCUAATT
	S2: CGAGGAGUCGAGCGUUGTGTT

Supporting Figure 1

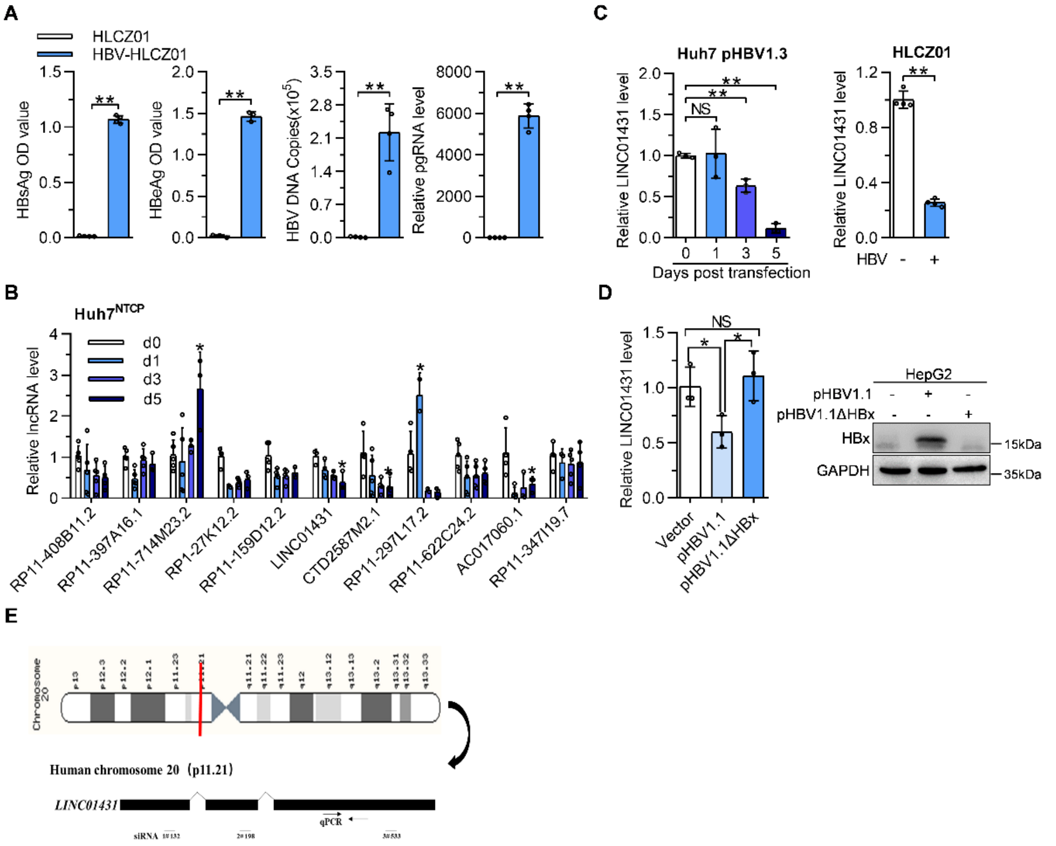

Supporting Figure 1. The level of LINC01431 is negatively correlated with HBV replication. (A) HLCZ01 cells were cultured with or without HBV infection for 5 days. The HBV antigens and HBV-DNA in the supernatant were detected using ELISA and RT-qPCR, respectively. (B) RT-qPCR analysis of LINC01431 RNA in HBV-infected Huh7^{NTCP} cells. (C) RT-qPCR analysis of LINC01431 RNA in pHBV1.3-transfected Huh7 and HBV-infected HLCZ01 cells. Huh7 cells were transfected with pHBV1.3, and the cells were harvested at indicated time post transfection (left). HLCZ01 cells were infected with HBV, the cells were harvested at day 5 post infection and used for RT-qPCR (right). (D) HepG2 cells were transfected with pHBV1.1 and pHBV1.1ΔHBx, and the level of LINC01431 was measured using RT-qPCR. (E) Schematic representation of exons and transcripts of *LINC01431* and its loci on human chromosome 20p11.21 in UCSC Genome browser (GRCh38.p13). For A-D, representative of 3 independent experiments. Data information: Data were presented as mean±SD and normalized to the control group. One-way ANOVA (D). Two-tail unpaired Student's *t*-tests; $^{*}P<0.05$; $^{**}P<0.01$; NS, no significance (A-C).

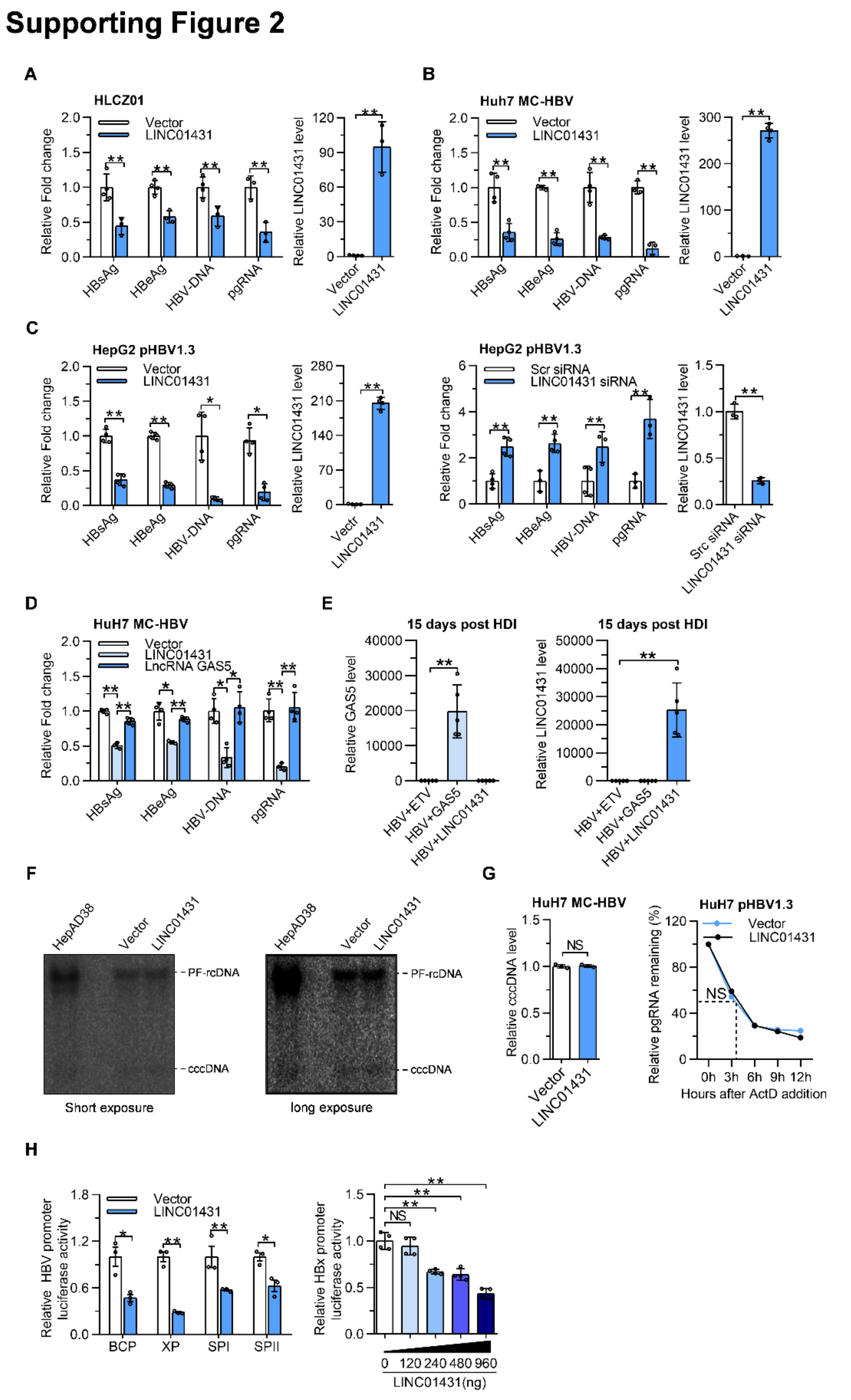

Supporting Figure 2. LINC01431 overexpression represses HBV replication. (A-B) LINC01431 overexpression was performed in HBV-infected HLCZ01 cells (A) and MC-HBV-transfected Huh7 cells (B), the levels of HBV antigens, HBV-DNA and pgRNA were detected using ELISA and RT-qPCR, respectively. (C) LINC01431 overexpression and knockdown were

performed in pHBV1.3-transfected HepG2 cells. The levels of HBV antigens, HBV-DNA and pgRNA were detected at day 3 post transfection. (D) LINC01431 and GAS5 were overexpressed in MC-HBV-transfected Huh7 cells, and the level of HBV antigens, HBV-DNA and pgRNA were detected using ELISA and RT-qPCR, respectively. (E) RT-qPCR analysis of GAS5 and LINC01431 RNA in HBV carrier mice liver tissues. (F) Southern blot was performed in HBV-infected HepaRG^{NTCP} cells at day 7 post infection (dpi). (G) RT-qPCR analysis of cccDNA (left) and the stability of pgRNA (right) in LINC01431-transfected Huh7 cells. (H) Luciferase reporter assay of HBV promoters in Huh7 cells. For all experiments, representative of 3 independent experiments. Data information: Data were presented as mean±SD and normalized to the control group. Two-tail unpaired Student's *t*-tests; $^{*}P<0.05$; $^{**}P<0.01$; NS, no significance (A-H).

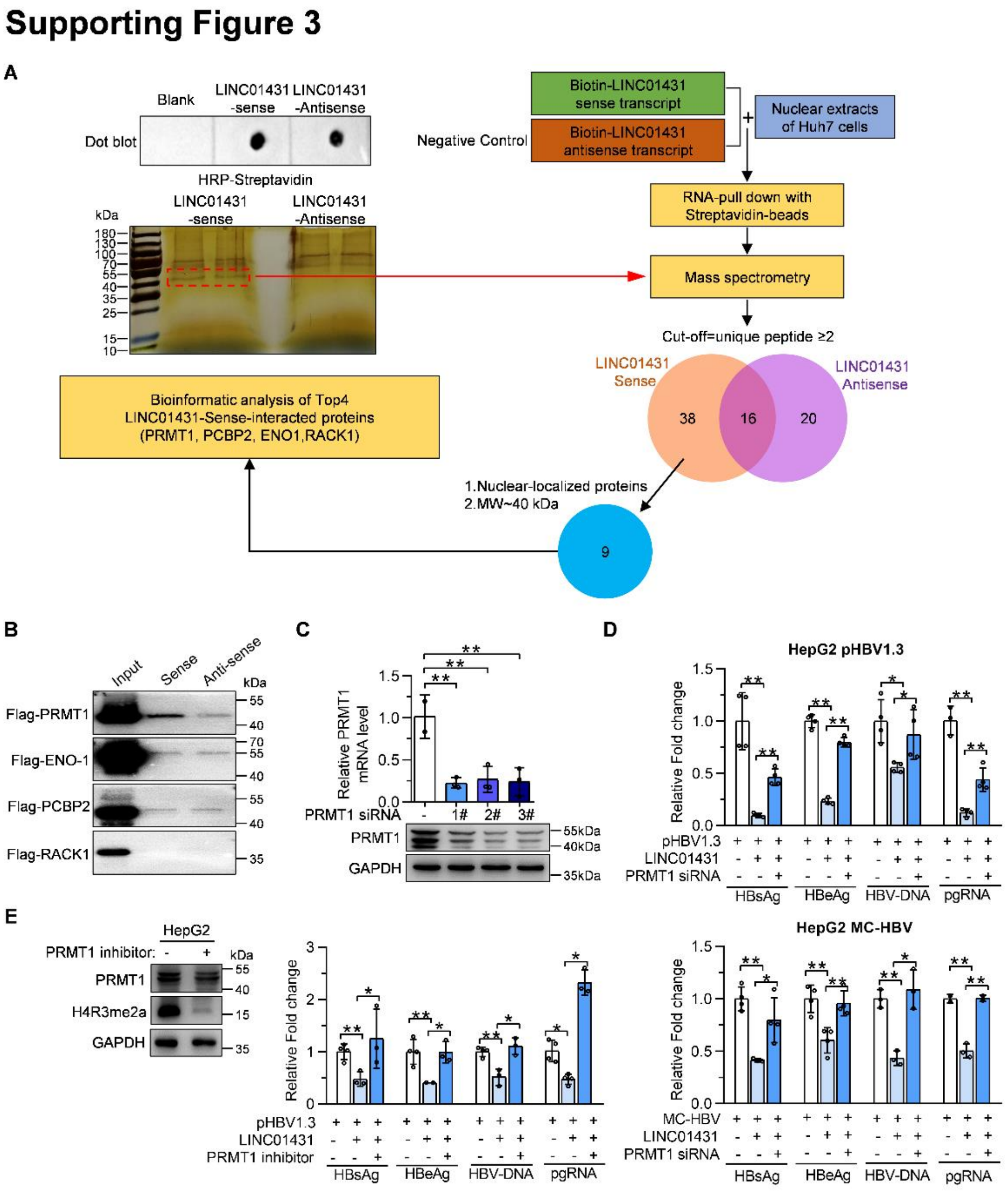

Supporting Figure 3. PRMT1 interacts with and mediates the inhibitory function of LINC01431 on HBV transcription. (A) RNA-pull down assay was performed using biotinylated LINC01431 RNA, and the retrieved proteins were subjected to SDS-PAGE followed by sliver staining. (B) RNA-pull down assay was performed using biotinylated LINC01431 RNA incubating with Huh7 extracts, and the retrieved proteins were subjected to immunoblot with indicated antibodies. (C) RT-qPCR and immunoblot analysis of PRMT1 in Huh7 cells after PRMT1 silencing. (D) Rescue assays were performed in pHBV1.3-transfected (upper) and MC-HBV-transfected (bottom) HepG2 cells after PRMT1 silencing. Levels of HBV antigens, HBV-DNA and pgRNA were detected at day 3 post transfection by ELISA and RT-qPCR, respectively. (E) Rescue assay was performed in HepG2 cells after treatment with the PRMT1 inhibitor C-7280948 (12.8 μM). The levels of H4R3me2a modification were detected by Western blot and the levels of HBV antigens, HBV-DNA and pgRNA were detected by ELISA and RT-qPCR, respectively. GAPDH served as the loading control. For all experiments, representative of 3 independent experiments. Data information: Data were presented as mean±SD and normalized

to the control group. One-way ANOVA (D, E). Two-tail unpaired Student’s *t*-tests; $^{*}P<0.05$; $^{**}P<0.01$ (C).

Supporting Figure 4

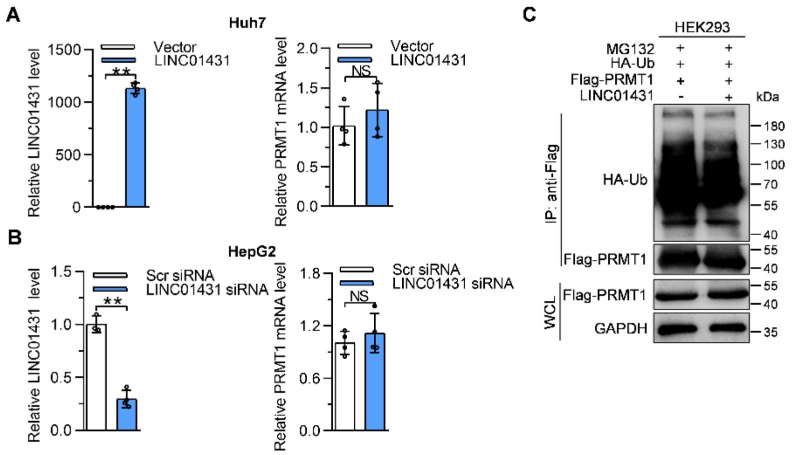

Supporting Figure 4. LINC01431 blocks the ubiquitination accumulation of PRMT1. (A, B) Huh7 cells were transfected with LINC01431 (A) and HepG2 cells were transfected with LINC01431 siRNA (B). The levels of LINC01431 and PRMT1 RNA were measured using RT-qPCR. (C) Ubiquitination of PRMT1 in LINC01431-transfected HEK293 cells. Flag-PRMT1 was immunoprecipitated with anti-Flag antibody, followed by immunoblotting with anti-HA antibody to detect ubiquitination level. WCL indicated the whole cell lysis. Flag-PRMT1 and GAPDH served as the loading control. For all experiments, representative of 3 independent experiments. Data information: Data were presented as mean±SD and normalized to the control group. Two-tail unpaired Student's *t*-tests; $^{*}P<0.05$; $^{**}P<0.01$; NS, no significance (A, B).

Supporting Figure 5

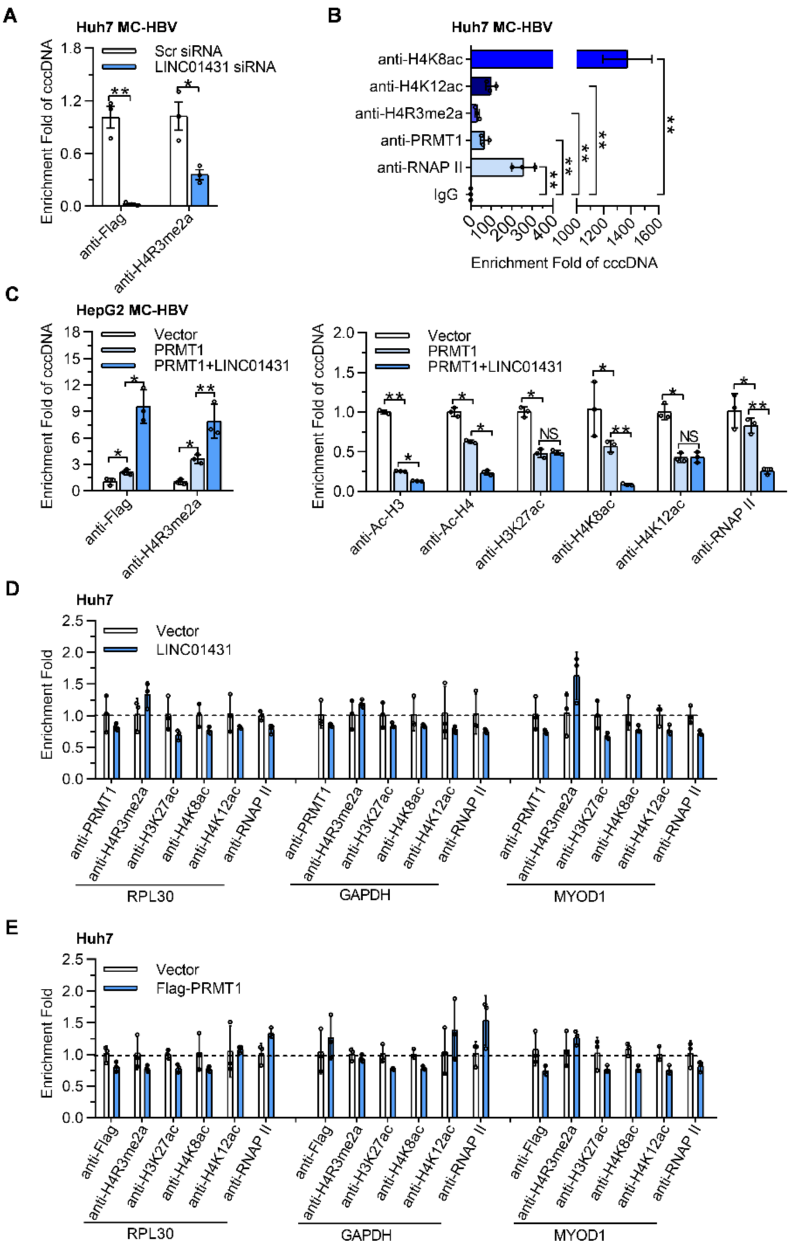

Supporting Figure 5. LINC01431 increases the occupancy of PRMT1 on cccDNA and induces cccDNA-associated histones hypoacetylation. (A) Huh7 cells were transfected with MC-HBV, Flag-PRMT1 and LINC01431 siRNA, and the enrichments of PRMT1 and H4R3me2a on cccDNA were detected at day 3 post transfection using indicated antibodies. (B) ChIP assay was performed in MC-HBV-transfected Huh7 cells, and the enrichments of epigenetic modifications on cccDNA were detected using indicated antibodies. (C) HepG2 cells were transfected with MC-HBV, Flag-PRMT1 and LINC01431, the enrichment of PRMT1, H4R3me2a and other epigenetic modifications on cccDNA were detected at day 3 post transfection. (D-E) ChIP assays were performed in LINC01431 (D) and PRMT1 (E)-transfected Huh7 cells. The enrichments of PRMT1, H4R3me2a and other epigenetic modifications on host genomic DNA were detected at day 3 post transfection with indicated antibodies. For all experiments,

representative of 3 independent experiments. Data information: Data were presented as mean±SD and normalized to the control group. One-way ANOVA (C). Two-tail unpaired Student's *t*-tests; [*]P<0.05; [**]P<0.01; NS, no significance (A, B, D, E).

Supporting Figure 6

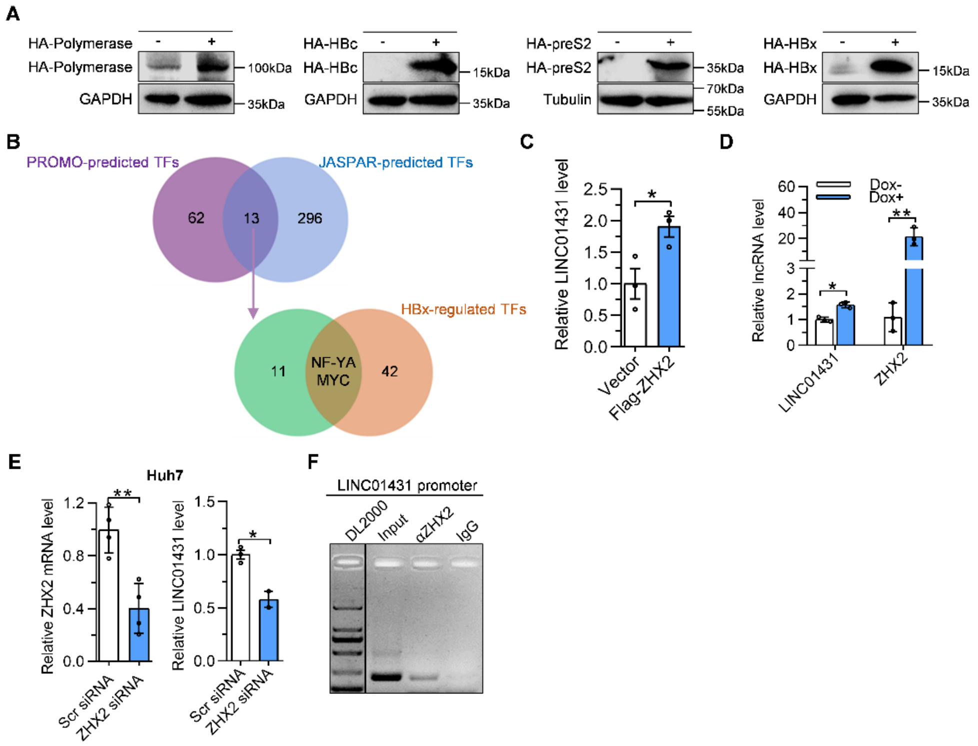

Supporting Figure 6. ZHX2 transcriptionally promotes LINC01431 expression. (A) Immunoblot analysis of HA-Polymerase, HA-HBc, HA-preS2 and HA-HBx in Huh7 cells. (B) Screening the transcription factors potentially modulating LINC01431 expression and as a target of HBx. (C)RT-qPCR and immunoblot analysis of LINC01431 RNA and ZHX2 in HepG2, respectively. (D) RT-qPCR analysis of LINC01431 and ZHX2 RNA in ZHX2 stably-integrated Bel7402 cells in the presence of doxycycline (4 μg/ml). (E) RT-qPCR analysis of LINC01431 and ZHX2 RNA in Huh7 cells after ZHX2 knockdown. (F) ChIP assay was performed in HepG2 cells, and the enrichment of ZHX2 on *LINC01431* promoter was visualized using gel electrophoresis. GAPDH，β-actin and Tubulin served as the loading control. For all experiments, representative of 3 independent experiments. Data information: Data were presented as mean±SD and normalized to the control group. Two-tail unpaired Student's *t*-tests; $^{*}P<0.05$; $^{**}P<0.01$ (C-D).
